# Supplementary material for: Impact of a national primary care pay-for-performance scheme on ambulatory care sensitive hospital admissions: a small-area analysis in England
Source: BMJ Open. 2020 Sep 9;10(9):e036046. doi: 10.1136/bmjopen-2019-036046 (PMC7482460; doi:10.1136/bmjopen-2019-036046)
Supplement: Supplementary data [file bmjopen-2019-036046supp001.pdf]

Supplementary Material

The impact of a national primary care pay-for-performance scheme on ambulatory care sensitive hospital admissions: A small area analysis in England

Supplemental Tables and Figures

Table S1. ICD codes used to define QOF incentivised ACSC hospital admissions

Table S2. QOF indicators included in the aggregate performance scores and their changes over time

Table S3 - Effects of QOF overall population achievement on hospital admissions for QOF incentivised ACSCs

Table S4 - Effects of QOF Population Achievement on age-adjusted hospital admissions for QOF incentivised ACSCs in 2015/16

Figure S1: Rates of hospital admissions for incentivised ACSCs per 100,000 people, 2006 – 2015 by condition type.

Figure S2: Age-adjusted rates of hospital admissions for incentivized ACSCs per 100,000 people in 2015.

Figures S3:S12 – Regional Spatial Maps of hospital admissions for QOF incentivised ACSCs at the LSOA level, 2015/16.

Figures S13:S22 – Regional Spatial Maps of Population Achievement for QOF incentivised ACSCs at the LSOA level, 2015/16.

Grigoroglou C, *et al. BMJ Open* 2020; 10:e036046. doi: 10.1136/bmjopen-2019-036046

Classification of ACSC hospital admissions for QOF incentivised conditions.

In HES, all admission data are related to episodes rather than persons as some individuals may have been admitted in hospitals multiple times over one calendar year and these presentations will be recorded as separate admissions. Classification of ACSC hospital admissions for the study used the International Classification of Diseases, 10th edition (ICD-10) and included all hospital admissions with primary diagnosis related to one of the 9 QOF incentivised conditions examined in this study. The specific 9 conditions were chosen based on the list of ACSCs which are used for measuring system performance in the NHS (1) plus a small number of diagnoses adopted from a previous study. (1) We also included complications of diabetes associated with hypoglycaemia. (2)

Table S1: ICD codes used to define QOF incentivised ACSC hospital admissions

J45, J46	Asthma
I20, I24.0, I24.8, I24.9, I25	Coronary Heart Disease
J20, J41, J42, J43, J44, J47	Chronic Obstructive Pulmonary Disease
E10.0–E10.8, E11.0–E11.8, E13.0–E13.8, E14.0–E14.8	Diabetes
E162	Diabetes (hypoglycaemic)
G40, G41	Epilepsy
I11.0, I13.0, I50, J81	Heart Failure
I10, I11.9	Hypertension
I61 I62 I63 I64 I66 I672 I698 R470	Stroke

Grigoroglou C, et al. BMJ Open 2020; 10:e036046. doi: 10.1136/bmjopen-2019-036046

Figure S1: Rates of hospital admissions for incentivised ACSCs per 100,000 people, 2006 – 2015 by condition type.

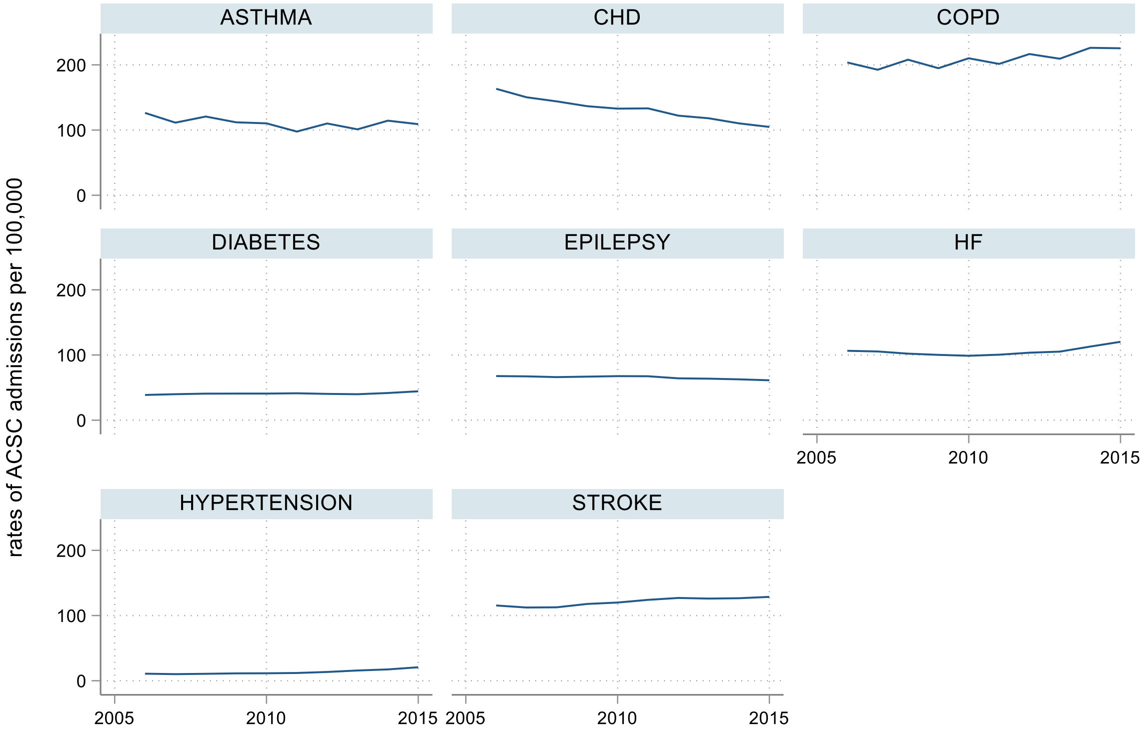

Description of indicators included in the composite measure of quality of care

Several indicators were used over the study period (2006 - 2015) and were aggregated into a single score. Although these indicators have been revised or rephrased many times over the years, their underlying aim has consistently remained a) to identify patients with symptoms of each respective condition b) to provide assessment and monitoring to those patients. Throughout the years of the scheme, some indicators were dropped while others were revised. When an indicator was dropped from the scheme, population achievement was calculated for each year from the remaining indicators. When an indicator was revised, the revision was concerned with the time span that measurement or treatment was taken or provided (e.g. for some indicators the time span was specified for 15 months but after the revision the time span was reduced to 12 months). In the composite measure we included all recording/measurement and treatment indicators related to the conditions outlines in table 1.

Table S2: OOF indicators included in the aggregate performance scores and their changes over time

Name	Year*	Type	Definition	Lt †	Ut †	P†
ASTHMA 3	3-8	Measurement	The percentage of patients with asthma between the ages of 14 and 19 in whom there is a record of smoking status in the previous 15 months	40	80	6
ASTHMA 6	3-8	Measurement	The percentage of patients with asthma who have had an asthma review in the last 15 months	40	70	20
ASTHMA 8	3-9	Measurement	The percentage of patients aged eight and over diagnosed as having asthma from 1 April 2006 with measures of variability or reversibility	40	80	15
ASTHMA 9	9	Measurement	The percentage of patients with asthma who have had an asthma review in the preceding 15 months that includes an assessment of asthma control using the 3 RCP questions	45	70	20
ASTHMA 10	9	Measurement	The percentage of patients with asthma between the ages of 14 and 19 years in whom there is a record of smoking status in the preceding 15 months	45	80	6
ASTHMA 002	10-12	Measurement	The percentage of patients aged 8 or with asthma (diagnosed on or after 1 April 2006) on the register with measures of variability or reversibility recorded between 3 months before and anytime after diagnosis	45	80	15
ASTHMA 003	10-12	Measurement	The percentage of patients with asthma on the register who have had an asthma review in the preceding 12 months that includes an assessment of asthma control using the 3 RCP questions	45	70	20
			The percentage of patients with asthma aged 14 or over and who have not attained the age of 20 on the register in whom there is a record of smoking	45	80	6

Grigoroglou C, *et al. BMJ Open* 2020; 10:e036046. doi: 10.1136/bmjopen-2019-036046

ASTHMA 004	10-12	Measurement	status in the preceding 12 months			
CHD 2	3-7	Measurement	The percentage of patients with newly diagnosed angina (diagnosed after 01/04/03) who are referred for exercise testing and/or specialist assessment	40	90	7
CHD 13	8-9	Measurement	For patients with newly diagnosed angina (diagnosed after 1 April 2011), the percentage who are referred for specialist assessment	40	90	7
CHD 5	3-7	Measurement	The percentage of patients with coronary heart disease whose notes have a record of blood pressure in the previous 15 months	40	90	7
CHD 6	3-5	Outcome	The percentage of patients with coronary heart disease, in whom the last blood pressure reading (measured in the last 15 months) is 150/90 or less	40	70	19
CHD 6	6-7	Outcome	The percentage of patients with coronary heart disease, in whom the last blood pressure reading (measured in the last 15 months) is 150/90 or less	40	70	17
CHD 6	8	Outcome	The percentage of patients with coronary heart disease, in whom the last blood pressure reading (measured in the last 15 months) is 150/90 or less	40	71	17
CHD 6	9	Outcome	The percentage of patients with coronary heart disease, in whom the last blood pressure reading (measured in the last 15 months) is 150/90 or less	40	75	17
CHD 7	3-7	Measurement	The percentage of patients with coronary heart disease whose notes have a record of total cholesterol in the previous 15 months	40	90	7
CHD 8	3-8	Outcome	The percentage of patients with coronary heart disease whose last measured total cholesterol (measured in the last 15 months) is 5 mmol/l or less	40	70	17
CHD 8	9	Outcome	The percentage of patients with coronary heart disease whose last measured total cholesterol (measured in the last 15 months) is 5 mmol/l or less	44	70	17
CHD 9	3-8	Treatment	The percentage of patients with coronary heart disease with a record in the last 15 months that aspirin, an alternative anti- platelet therapy, or an anti-coagulant is being taken (unless a contraindication or side effects are recorded)	40	90	7
CHD 9	9	Treatment	The percentage of patients with coronary heart disease with a record in the last 15 months that aspirin, an alternative anti- platelet therapy, or an anti-coagulant is being taken (unless a contraindication or side effects are recorded)	50	90	7

CHD 10	3-8	Treatment	The percentage of patients with coronary heart disease who are currently treated with a beta blocker (unless a contraindication or side-effects are recorded)	40	60	7
CHD 10	9	Treatment	The percentage of patients with coronary heart disease who are currently treated with a beta blocker (unless a contraindication or side-effects are recorded)	40	65	7
CHD 11	3-7	Treatment	The percentage of patients with a history of myocardial infarction (diagnosed after 1 April 2003) who are currently treated with an ACE inhibitor	40	80	7
CHD 14	8	Treatment	The percentage of patients with a history of myocardial infarction (from 1 April 2011) currently treated with an ACE inhibitor (or ARB if ACE intolerant), aspirin or an alternative anti-platelet therapy, beta blocker or statin (unless a contraindication or side effects are recorded)	40	80	10
CHD 14	9	Treatment	The percentage of patients with a history of myocardial infarction (from 1 April 2011) currently treated with an ACE inhibitor (or ARB if ACE intolerant), aspirin or an alternative anti-platelet therapy, beta blocker or statin (unless a contraindication or side effects are recorded)	45	80	10
CHD 12	3-8	Treatment	The percentage of patients with coronary heart disease who have a record of influenza vaccination in the preceding 1 September to 31 March	40	90	7
CHD 12	9	Treatment	The percentage of patients with coronary heart disease who have a record of influenza vaccination in the preceding 1 September to 31 March	50	90	7
CHD 002	10-12	Outcome	The percentage of patients with coronary heart disease, in whom the last blood pressure reading (measured in the last 12 months) is 150/90mmHg or less	53	93	17
CHD 003	10	Outcome	The percentage of patients with coronary heart disease whose last measured total cholesterol (measured in the last 12 months) is 5 mmol/l or less	45	85	17
CHD 005	10-12	Treatment	The percentage of patients with coronary heart disease with a record in the last 12 months that aspirin, an alternative anti-platelet therapy, or an anti-coagulant is being taken (unless a contraindication or side effects are recorded)	56	96	7
CHD 006	10-12	Treatment	The percentage of patients with coronary heart disease with a record in the last 12 months that aspirin, an alternative anti-platelet therapy, or an anti-coagulant is being taken (unless a contraindication or side effects are recorded)	60	100	10
CHD 004	10	Treatment	The percentage of patients with coronary heart disease who have a record of influenza vaccination in the preceding 1 September to 31 March	56	96	7

CHD 007	11-12	Treatment	The percentage of patients with coronary heart disease who had influenza vaccination in the preceding 1 August to 31 March	56	96	7
COPD 8	3-8	treatment	The percentage of patients with COPD who have had influenza immunisation in the preceding 1 September to 31 March	40	85	6
COPD 8	9	treatment	The percentage of patients with COPD who have had influenza immunisation in the preceding 1 September to 31 March	45	85	6
COPD 10	3-8	Measurement	The percentage of patients with COPD with a record of FeV1 in the previous 15 months	40	70	7
COPD 10	9	Measurement	The percentage of patients with COPD with a record of FeV1 in the previous 15 months	40	75	7
COPD 11	3-5	Treatment	The percentage of patients with COPD receiving inhaled treatment in whom there is a record that inhaler technique has been checked in the previous 15 months	40	90	7
COPD 13	6-9	Treatment	The percentage of patients with COPD who have had a review, undertaken by a healthcare professional, including an assessment of breathlessness using the MRC dyspnoea score in the preceding 15 months.	50	90	9
COPD 12	3-7	Measurement	The percentage of all patients with COPD diagnosed after 1st April 2008 in whom the diagnosis has been confirmed by post bronchodilator spirometry	40	80	5
COPD 15	8	Measurement	The percentage of all patients with COPD diagnosed after 1 April 2011 in whom the diagnosis had been confirmed by post bronchodilator spirometry	40	80	5
COPD 15	9	Measurement	The percentage of all patients with COPD diagnosed after 1 April 2011 in whom the diagnosis had been confirmed by post bronchodilator spirometry	45	80	5
COPD 002	10-12	Measurement	The percentage of all patients with COPD diagnosed after 1 April 2011 in whom the diagnosis had been confirmed by post bronchodilator spirometry between 3 months before and 12 months after entering on to the register	45	80	5
COPD 004	10-12	Measurement	The percentage of patients with COPD with a record of FeV1 in the preceding 12 months	40	75	7
COPD 003	10-12	Treatment	The percentage of patients with COPD who have had a review, undertaken by a healthcare professional, including an assessment of breathlessness using the MRC dyspnoea score in the preceding 12 months.	50	90	9
COPD 006	10	Treatment	The percentage of patients with COPD who have had influenza immunisation in the preceding 1 September to 31 March	57	97	6

Grigoroglou C, *et al. BMJ Open* 2020; 10:e036046. doi: 10.1136/bmjopen-2019-036046

COPD 007	11-12	Treatment	The percentage of patients with COPD who have had influenza immunisation in the preceding 1 August to 31 March	57	97	6
COPD 005	10-12	Measurement	The percentage of patients with COPD and MRC dyspnoea grade ≥3 at any timein the preceding 12 months, with a record of oxygen saturation value within the preceding 12 months	40	90	5
DM 2	3-8	Measurement	The percentage of patients with diabetes whose notes record BMI in the previous 15 months	40	90	3
DM 2	9	Measurement	The percentage of patients with diabetes whose notes record BMI in the previous 15 months	50	90	3
DM 5	3-7	Measurement	The percentage of diabetic patients who have a record of HbA1c or equivalent in the previous 15 months	40	90	3
DM 7	3-7	Outcome	The percentage of patients with diabetes in whom the last HbA1C is 10 or less (or equivalent test / reference range depending on local laboratory) in last 15 months	40	90	11
DM 9	3-7	Measurement	The percentage of patients with diabetes with a record of presence or absence of peripheral pulses in the previous 15 months	40	90	3
DM 29	8	Measurement	The percentage of patients with diabetes with a record of a foot examination and risk classification: 1) low risk (normal sensation, palpable pulses), 2) increased risk (neuropathy or absent pulses), 3) high risk (neuropathy or absent pulses plus deformity or skin changes or previous ulcer) or 4) ulcerated foot within the preceding 15 months	40	90	4
DM 29	9	Measurement	The percentage of patients with diabetes with a record of a foot examination and risk classification: 1) low risk (normal sensation, palpable pulses), 2) increased risk (neuropathy or absent pulses), 3) high risk (neuropathy or absent pulses plus deformity or skin changes or previous ulcer) or 4) ulcerated foot within the preceding 15 months	50	90	4
DM 10	3-8	Measurement	The percentage of patients with diabetes with a record of neuropathy testing in the previous 15 months	40	90	3
DM 10	9	Measurement	The percentage of patients with diabetes with a record of neuropathy testing in the previous 15 months	50	90	3
DM 11	3-7	Measurement	The percentage of patients with diabetes who have a record of the blood pressure in the past 15 months	40	90	3

DM 12	3-7	Outcome	The percentage of patients with diabetes in whom the last blood pressure is 145/85 or less	40	60	18
DM30	8	Outcome	The percentage of patients with diabetes in whom the last blood pressure is 150/90 or less	40	71	8
DM30	9	Outcome	The percentage of patients with diabetes in whom the last blood pressure is 150/90 or less	45	71	8
DM 13	3-8	Measurement	The percentage of patients with diabetes who have a record of micro-albuminuria testing in the previous 15 months (exception reporting for patients with proteinuria)	40	90	3
DM 005	9	Measurement	The percentage of patients with diabetes on the register who have a record of an albumin:creatinine ratio test in the previous 12 months	50	90	3
DM 15	3-8	Treatment	The percentage of patients with diabetes with proteinuria or micro-albuminuria who are treated with ACE inhibitors (or A2 antagonists)	40	80	3
DM 15	9	Treatment	The percentage of patients with diabetes with proteinuria or micro-albuminuria who are treated with ACE inhibitors (or A2 antagonists)	45	80	3
DM 16	3-7	Measurement	The percentage of patients with diabetes who have a record of total cholesterol in the previous 15 months	40	90	3
DM 17	3-7	Outcome	The percentage of patients with diabetes whose last measured total cholesterol within previous 15 months is 5 or less	40	70	6
DM 17	8	Outcome	The percentage of patients with diabetes whose last measured total cholesterol within previous 15 months is 5 or less	40	70	6
DM 17	9	Outcome	The percentage of patients with diabetes whose last measured total cholesterol within previous 15 months is 5 or less	40	75	6
DM 18	3-8	Treatment	The percentage of patients with diabetes who have had influenza immunisation in the preceding 1 September to 31 March	40	85	3
DM 18	9	Treatment	The percentage of patients with diabetes who have had influenza immunisation in the preceding 1 September to 31 March	45	85	3
DM 20	3-6	Outcome	The percentage of patients with diabetes in whom the last HbA1C is 7.5 or less (or equivalent test / reference range depending on local laboratory) in the previous 15 months	40	50	17
DM 23	7	Outcome	The percentage of patients with diabetes in whom the last HbA1c is 7 or less (or equivalent test/reference range depending on local laboratory) in the previous 15 months.	40	50	17

DM 26	8-9	Outcome	The percentage of patients with diabetes in whom the last IFCC-HbA1c is 75 mmol/mol (equivalent to HbA1c of 9% in DCCT values) or less (or equivalent test/reference range depending on local laboratory) in the previous 15 months.	40	90	10
DM 21	3-9	Measurement	The percentage of patients with diabetes who have a record of retinal screening in the previous 15 months	40	90	5
DM 22	3-8	Measurement	The percentage of patients with diabetes who have a record of estimated glomerular filtration rate (eGFR) or serum creatinine testing in the previous 15 months	40	90	3
DM 22	9	Measurement	The percentage of patients with diabetes who have a record of estimated glomerular filtration rate (eGFR) or serum creatinine testing in the previous 15 months	50	90	3
DM 24	6-7	Outcome	The percentage of patients with diabetes in whom the last HbA1c is 8 or less (or equivalent test/reference range depending on local laboratory) in the previous 15 months.	40	70	8
DM 27	8	Outcome	The percentage of patients with diabetes in whom the last IFCC-HbA1c is 64 mmol/mol (equivalent to HbA1c of 8% in DCCT values) or less (or equivalent test/reference range depending on local laboratory) in the previous 15 months.	40	70	8
DM 27	8	Outcome	The percentage of patients with diabetes in whom the last IFCC-HbA1c is 64 mmol/mol (equivalent to HbA1c of 8% in DCCT values) or less (or equivalent test/reference range depending on local laboratory) in the previous 15 months.	45	70	8
DM 31	8	Outcome	The percentage of patients with diabetes in whom the last blood pressure is 140/80 or less	40	60	10
DM 007	10-12	Outcome	The percentage of patients with diabetes on the register in whom the last IFCC-HbA1c is 59 mmol/mol (equivalent to HbA1c of 7.5% in DCCT values) or less (or equivalent test/reference range depending on local laboratory) in the previous 12 months.	35	75	17
DM 009	10-12	Outcome	The percentage of patients with diabetes on the register in whom the last IFCC-HbA1c is 75 mmol/mol (equivalent to HbA1c of 9% in DCCT values) or less (or equivalent test/reference range depending on local laboratory) in the previous 12 months.	52	92	10

Grigoroglou C, *et al. BMJ Open* 2020; 10:e036046. doi: 10.1136/bmjopen-2019-036046

DM 011	10	Recording/measurement	The percentage of patients with diabetes on the register who have a record of retinal screening in the previous 12 months	50	90	5
DM 012	10-12	Recording/measurement	The percentage of patients with diabetes with a record of a foot examination and risk classification: 1) low risk (normal sensation, palpable pulses), 2) increased risk (neuropathy or absent pulses), 3) high risk (neuropathy or absent pulses plus defomity or skin changes in previous ulcer) or 4) ulcerated foot within the preceding 12 months	50	90	5
DM 002	10-12	Outcome	The percentage of patients with diabetes on the register in whom the last blood pressure (measured in the last 12 months) is 150/90 or less	53	93	8
DM 005	10	Recording/measurement	The percentage of patients with diabetes on the register who have a record of an albumin:creatinine ratio test in the previous 12 months	50	90	3
DM 006	10-12	Treatment	The percentage of patients with diabetes on the register with a diagnosis of nephropathy (clinical proteinuria) or micro-albuminuria who are treated with ACE inhibitors (or ARBs)	57	97	3
DM 004	10-12	Outcome	The percentage of patients with diabetes on the register whose last measured total cholesterol within previous 12 months is 5 or less	40	75	6
DM 010	10	Treatment	The percentage of patients on the register who have had influenza immunisation in the preceding 1 September to 31 March.	55	95	3
DM 017	11-12	Treatment	The percentage of patients on the register who have had influenza immunisation in the preceding 1 August to 31 March.	55	95	3
DM 008	10-12	Outcome	The percentage of patients with diabetes on the register in whom the last IFCC-HbA1c is 64 mmol/mol (equivalent to HbA1c of 8% in DCCT values) or less (or equivalent test/reference range depending on local laboratory) in the previous 12 months.	43	83	8
DM 003	10-12	Outcome	The percentage of patients with diabetes on the register in whom the last blood pressure reading (measured in the last 12 months) is 140/80 or less	38	78	10

Grigoroglou C, *et al. BMJ Open* 2020; 10:e036046. doi: 10.1136/bmjopen-2019-036046

DM 013	10	Recording/measurement	The percentage of patients with diabetes, on the register, who have a record of a dietary review by a suitably competent professional in the preceding 12 months	40	90	3
DM 014	10-12	Treatment	The percentage of patients with diabetes, on the register, in the preceding 1 April to 31 March who have a record of being referred to a structured education programme within 9 months after entry on to the diabetes register	40	90	11
DM 015	10	Recording/measurement	The percentage of male patients with diabetes, on the register, with a record of being asked about erectile dysfunction in the preceding 12 months	40	90	4
DM 016	10	Treatment	The percentage of male patients with diabetes, on the register, with a record of erectile dysfunction with a record of advice and assessment of contributory factors and treatment options in the preceding 12 months	40	90	6
EPILEPSY 6	3-8	Measurement	The percentage of patients age 16 and over on drug treatment for epilepsy who have a record of seizure frequency in the previous 15 months	40	90	4
EPILEPSY 7	3-7	Measurement	The percentage of patients age 18 and over on drug treatment for epilepsy who have a record of medication review involving the patient and/or carer in the previous 15 Months	40	90	4
EPILEPSY 8	3-8	Outcome	The percentage of patients age 18 and over on drug treatment for epilepsy who have been seizure free for last 12 months recorded in last 15 months	40	70	6
EPILEPSY 9	8	Measurement	The percentage of women under the age of 55 years who are taking antiepileptic drugs who have a record of information and counselling about contraception, conception and pregnancy in the preceding 15 months	40	90	3
EPILEPSY 6	9	Measurement	The percentage of patients age 16 and over on drug treatment for epilepsy who have a record of seizure frequency in the previous 15 months	50	90	4
EPILEPSY 8	9	Outcome	The percentage of patients age 18 and over on drug treatment for epilepsy who have been seizure free for last 12 months recorded in last 15 months	45	70	6
EPILEPSY 9	9	Measurement	The percentage of women under the age of 55 years who are taking antiepileptic drugs who have a record of information and counselling about contraception, conception and pregnancy in the preceding 15 months	50	90	3

EP 002	10	Measurement	The percentage of patients age 18 and over on drug treatment for epilepsy who have been seizure free for last 12 months recorded in last 12 months	45	70	6
EP 003	10	Measurement	The percentage of women under the age of 55 years who are taking antiepileptic drugs who have a record of information and counselling about contraception, conception and pregnancy in the preceding 12 months	50	90	3
HF 2	3-8	Measurement	The percentage of patients with a diagnosis of heart failure (diagnosed after 1 April 2006) which has been confirmed by an ecocardiogram or by a specialist	40	90	6
HF 3	3-8	Treatment	The percentage of patients with a current diagnosis of heart failure due to LVD who are currently treated with an ACE inhibitor or Angiotensin Receptor Blocker who can tolerate therapy and for whom there is no contraindication	40	80	10
HF 4	6-8	Treatment	The percentage of patients with a current diagnosis of heart failure due to LVD who are currently treated with an ACE inhibitor or Angiotensin Receptor, who are additionally treated with a beta-blocker licensed for heart failure, or recorded as intolerant to or having a contraindication to beta-blockers.	40	60	9
HF 2	9	Measurement	The percentage of patients with a diagnosis of heart failure (diagnosed after 1 April 2006) which has been confirmed by an ecocardiogram or by a specialist	50	90	6
HF 2	9	Measurement	The percentage of patients with a diagnosis of heart failure (diagnosed after 1 April 2006) which has been confirmed by an ecocardiogram or by specialist assessment 3 months before or 12 months after entering on the register	50	90	6
HF 3	9	Treatment	The percentage of patients with a current diagnosis of heart failure due to LVD who are currently treated with an ACE inhibitor or Angiotensin Receptor Blocker who can tolerate therapy and for whom there is no contraindication	45	80	10
HF 3	10-12	Treatment	The percentage of patients with a diagnosis of heart failure (diagnosed after 1 April 2006) which has been confirmed by an ecocardiogram or by specialist assessment 3 months before or 12 months after entering on the register	60	100	10
			The percentage of patients with a current diagnosis of heart failure due to LVD who are currently treated with an ACE inhibitor or Angiotensin Receptor, who are additionally treated with a beta-blocker licensed for heart			

HF 4	9	Treatment	failure, or recorded as intolerant to or having a contraindication to beta-blockers.	40	65	9
HF 4	10-12	Treatment	In those patients with a current diagnosis of heart failure due to LVD who are currently treated with an ACE-i or ARB, the percentage of patients who are additionally currently treated with a beta-blocker licensed for heart failure.	40	65	9
BP 4	3-5	Measurement	The percentage of patients with hypertension in which there is a record of the blood pressure in the past 9 months	40	90	20
BP 4	6-7	Measurement	The percentage of patients with hypertension in which there is a record of the blood pressure in the past 9 months	40	90	18
BP 4	8	Measurement	The percentage of patients with hypertension in which there is a record of the blood pressure in the past 9 months	40	90	16
BP 4	9	Measurement	The percentage of patients with hypertension in which there is a record of the blood pressure in the past 9 months	50	90	16
BP 5	3-8	Outcome	The percentage of patients with hypertension in whom the last blood pressure (measured in last 9 months) is 150/90 or less	40	70	57
BP 5	9	Outcome	The percentage of patients with hypertension in whom the last blood pressure (measured in last 9 months) is 150/90 or less	45	80	57
HYP 002	10	Outcome	The percentage of patients with hypertension in whom the last blood pressure reading (measured in last 9 months) is 150/90 or less	44	84	10
HYP 006	11-12	Outcome	The percentage of patients with hypertension in whom the last blood pressure reading (measured in last 12 months) is 150/90 or less	45	80	20
HYP 003	10	Outcome	The percentage of patients aged 79 or under with hypertension in whom the last blood pressure reading (measured in last 9 months) is 140/90 or less	40	80	50
HYP 004	10	Measurement	The percentage of patients with hypertension aged 16 or over and who have not attained the age of 75 in whom there is an assessment of physical activity, using GPPAQ, in the preceding 12 months	40	80	5
HYP 005	10	Treatment	The percentage of patients with hypertension aged 16 or over and who have not attained the age of 75 who score 'less than active' on GPPAQ in the preceding 12 months, who have a record of a brief intervention in the preceding 12 months	40	80	6

STROKE 5	3-7	Measurement	The percentage of patients with TIA or stroke who have a record of blood pressure in the notes in the preceding 15 months	40	90	2
STROKE 6	3-7	Outcome	The percentage of patients with a history of TIA or stroke in whom the last blood pressure reading (measured in the last 15 months) is 150/90 or less	40	70	5
STROKE 6	8	Outcome	The percentage of patients with a history of TIA or stroke in whom the last blood pressure reading (measured in the last 15 months) is 150/90 or less	40	71	5
STROKE 6	9	Outcome	The percentage of patients with a history of TIA or stroke in whom the last blood pressure reading (measured in the last 15 months) is 150/90 or less	40	75	5
STROKE 7	3-8	Measurement	The percentage of patients with TIA or stroke who have a record of total cholesterol in the last 15 months	40	90	2
STROKE 7	3-8	Measurement	The percentage of patients with TIA or stroke who have a record of total cholesterol in the last 15 months	50	90	2
STROKE 8	3-8	Outcome	The percentage of patients with TIA or stroke whose last measured total cholesterol (measured in the last 15 months) is 5 mmol/l or less	40	60	5
STROKE 8	3-8	Outcome	The percentage of patients with TIA or stroke whose last measured total cholesterol (measured in the last 15 months) is 5 mmol/l or less	40	65	5
STROKE 10	3-8	Treatment	The percentage of patients with TIA or stroke who have had influenza immunisation in the preceding 1 September to 31 March	40	85	2
STROKE 10	3-8	Treatment	The percentage of patients with TIA or stroke who have had influenza immunisation in the preceding 1 September to 31 March	45	85	2
STROKE 12	3-8	Treatment	The percentage of patients with a stroke shown to be non- haemorrhagic, or a history of TIA, who have a record that an anti-platelet agent (aspirin, clopidogrel, dipyridamole or a combination), or an anti-coagulant is being taken (unless a contraindication or side-effects are recorded)	40	90	4
STROKE 12	3-8	Treatment	The percentage of patients with a stroke shown to be non- haemorrhagic, or a history of TIA, who have a record that an anti-platelet agent (aspirin, clopidogrel, dipyridamole or a combination), or an anti-coagulant is being taken (unless a contraindication or side-effects are recorded)	50	90	4
STROKE 11	3-4	Measurement	The percentage of new patients with a stroke who have been referred for further investigation	40	80	2
			The percentage of new patients with a stroke or TIA who have been referred for further investigation	40	80	2

STROKE 13	5-8	Measurement	between 3 months before or 1 month after the date of the latest recorded stroke or TIA			
STROKE 13	9	Measurement	The percentage of new patients with a stroke or TIA who have been referred for further investigation between 3 months before or 1 month after the date of the latest recorded stroke or TIA	45	80	2
STIA 002	10	Measurement	The percentage of new patients with a stroke or TIA (diagnosed on or after 1 April 2008) who have a record of a referral for further investigation between 3 months before or 1 month after the date of the latest recorded stroke or TIA	45	80	2
STIA 003	10-12	Outcome	The percentage of patients with a history of TIA or stroke in whom the last blood pressure reading (measured in the last 12 months) is 150/90 or less	40	75	5
STIA 004	10	Measurement	The percentage of patients with TIA or stroke who have a record of total cholesterol in the last 12 months	50	90	2
STIA 005	10	Outcome	The percentage of patients with TIA or stroke whose last measured total cholesterol (measured in the last 12 months) is 5 mmol/l or less	40	65	5
STIA 007	10-12	Treatment	The percentage of patients with a stroke shown to be non-haemorrhagic, or a history of TIA, who have a record that aspirin, an alternative anti-platelet therapy, or an anti-coagulant is being taken	57	97	4
STIA 006	10	Treatment	The percentage of patients with TIA or stroke who have had influenza immunisation in the preceding 1 August to 31 March	55	95	2
STIA 002	11-12	Measurement	The percentage of new patients with a stroke or TIA (diagnosed on or after 1 April 2014) who have a record of a referral for further investigation between 3 months before or 1 month after the date of the latest recorded stroke or the first TIA	45	80	2
STIA 006	11-12	Treatment	The percentage of patients with TIA or stroke who have had influenza immunisation in the preceding 1 August to 31 March	55	95	2

* Corresponds to year of QOF, with year 1 being 2004/5, 2 to 2005/6 and so on up to year 12 (2015/16). This study considers QOF years 3-12.

† Lt: Lower threshold; Ut: Upper threshold; P: points indicator is worth (1 point ≈ £126). Also, $P = \min\{(Ut - Lt),\ (RA - Lt)/(Ut - Lt)\}$ where RA is the practice reported achievement (excluding exception reported patients) under the Quality and Outcomes Framework.

Description of population and area characteristics used in statistical models

English Indices of Deprivation

Area deprivation, as measured by the latest update of the Index of Multiple Deprivation (IMD) (i.e.2015) was available at the 2011 Lower Super Output (LSOA) level. The IMD is the most complete and widely used approach to quantify relative deprivation and affluence for small areas in England. The IMD measures relative levels of deprivation for all the 32,844 LSOAs in England on a continuous scale of deprivation where most of the indicators are based on 2012 statistics. It is a combined score of deprivation based on a total of 37 separate indicators that have been grouped into seven domains, each of which reflects a different aspect of deprivation experienced by individuals living in an area. The overall measure is calculated as a weighted mean across seven domains: income, employment, education and skills, health and disability, crime, barriers to housing and services, and living environment with different weights given to each domain. The Index of Multiple Deprivation is assigned to every small area in England and ranks them from 1 (most deprived area) to 32,844 (least deprived area). The IMD is widely used across central government to focus programmes on the most deprived areas. (3)

Lower Super Output Areas

The low geographical units to which the indices of deprivation are assigned are called lower super output areas (LSOAs) and they are designed to contain around 1500 inhabitants, on average. Following the 2011 census, there were 32,844 English LSOAs. (4) Urbanity information was also updated following the census, and we used a rural vs urban dichotomy for simplicity, with settlements with 10,000 people or more defined as urban. (5) Census-adjusted population estimates over time, by age groups and sex and for each English LSOA, were obtained from the Office of National Statistics. (6) Spatial coordinates for the 2011 LSOAs were obtained from the ONS open geography portal. We used digital vector boundaries generalised to 20 meters and clipped to the coastline to reduce size and improve visualisation. (7) To allow for comparisons within England, we organised LSOAs into 10 regions: North East, North West, Yorkshire & the Humber, East Midlands, West Midlands, East of England, London, South East Coast, South Central and South West.

Ethnicity

Ethnicity was taken from the 2011 census and was available at the LSOA level from the Nomis website. (8) Ethnicity classifies people according to their own perceived ethnic group and cultural background. The variable uses a harmonised country specific ethnic group question and the question is recommended when a show card is used in a face to face interview or self-completion survey (both paper and electronic).

Sex and population estimates

From the Office for National Statistics, we obtained mid-year population estimates for 2015 at the LSOA level. (6) The mid-year population estimates are the official set of population estimates for the UK and its constituent countries, the regions of England and Wales and local authorities. They are used directly as a base for other secondary population statistics, such as population projections, population estimates for the very old and population estimates for small geographical areas. A combination of registration, survey and administrative data are used to estimate the different components of population change. The data are provided for the whole population as well as by sex. We used the number of females in an LSOA in 2015 as recorded by ONS to calculate the percentage of females who were resident in each LSOA in 2015-2016.

Spatial Weighted analyses and attribution methodology

We used general practice level information from NHS Digital on several variables of interest to attribute it to our units of analyses, namely the LSOAs. We inputted the LSOA centroid coordinates (longitude and latitude) in R to create a 32,844x32,844 inverse distance matrix (in miles). This matrix features a detailed distance mapping of each LSOA with all other LSOAs and was used to quantify geographical proximity where nearby LSOAs have larger weights and also to generate prevalence and a measure for quality of care for all LSOAs from 2006-2007 to 2015-2016.

In 2014, NHS Digital published for the first time attribution datasets which linked general practice registers to LSOAs and vice versa. (9) We used the relevant version of the attribution dataset (i.e. from 2016) as a blueprint to generate annual attribution datasets starting from 2015-16 and going back to 2006-07. Of the 7856 practices in our analyses, 7625 (95·2%) were identified in the attribution dataset, 144 practices (3.70%)

had closed down or merged, while 87 new practices (1.1%) emerged. To calculate attribution rates for all years in order to subsequently quantify prevalence and quality of care we used regression modelling under various assumptions to obtain attribution estimates for previous years. For each LSOA, if two or more practices were linked to it, we fitted Poisson and negative binomial models with list size and distance to practice as predictors, and the model that was the best fit to the data was selected. If a practice was present both in the analyses and the attribution dataset, we adjusted the attributed population for practice' list size in the respective year, thus assuming a constant attribution rate over time. If a practice was present in our analyses but not in the attribution dataset, we generated estimates using the models selected in step 1 across all years. If a LSOA was served entirely by a single practice, we assumed that this was the case in previous years. Those practices that emerged after our baseline year were used only to model the 2015 attribution in the area. Redistribution of patients to the other active practices within each year was achieved according to the selected regression model. In the same manner, for those practices that closed down or merged, their patients were re-distributed to the years in which they were active, according to their characteristics. Finally, the attribution counts estimated in the previous steps across practices and within each year were used to generate the weighted mean estimates for prevalence and quality of care. The algorithm is available from the corresponding author. We assumed that the attribution rates remained constant over time, and we used this assumption to model the contribution of each practice; even the ones that had closed or merged by 2015. This method can possibly introduce uncertainty in the estimates which we could not include in the models because of methodological limitations. Even though the limitations and assumptions made for our approach could attenuate the relationship between quality of care and suicide, we would expect any strong relationship between our variables of interest to be detected as it was for example detected for prevalence of depression.

Spatial Maps

Digital vector boundaries for the 2011 LSOAs, generalised to 20 metres and clipped to the coastline to reduce size and improve visualisation, were obtained from the ONS open geography portal. (10) The vector boundaries were inputted in the Stata shp2dta command to calculate the centroid for each LSOA in the British National Grid format. (11) These were then converted from British National Grid easting and northing to longitude and latitude in degrees. (12)

Sensitivity analyses results

We conducted a sensitivity analyses under different assumptions to test the presence of The results from our sensitivity analyses were very similar to the results from the principal analysis and the coefficients had the same direction. First, due to the presence of many zeros we tested how a zero-inflated negative binomial (ZINB) fitted our data by running a regression with exactly the same covariates as our main model (i.e. negative binomial model presented in the main paper). The ZINB model assumes that the excess zero counts come from a logit or probit model and the remaining counts come from a negative binomial model. We modelled our counts as follows: We modelled excessive zeros using a logit model with the LSOA population as the only predictor and the remaining counts were modelled from the negative binomial model that we specified in the manuscript using deprivation, age, sex, rurality and ethnicity as predictors. The results from the zero inflated negative binomial regression indicate a very small association (0.993; 95CI 0.990, 0.995) between QOF Population Achievement and ACSC admissions for incentivised conditions. All other results were identical to the negative binomial regression mode. Over time our results indicated slightly smaller effect (0.998; 95CI 0.997, 0.999).

Grigoroglou C, *et al. BMJ Open* 2020; 10:e036046. doi: 10.1136/bmjopen-2019-036046

Table S3 - Effects of QOF overall population achievement on hospital admissions for QOF incentivised ACSCs

Year:	Negative Binomial Model	Zero Inflated Negative Binomial Model	Negative Binomial Model (Over time)	Zero Inflated Negative Binomial Model (Over time)
% Population achievement	0.993 (0.990, 0.995), <0.001 (0.001)	0.993 (0.990, 0.995), <0.001 (0.001)	0.998 (0.997, 0.999), <0.001 (0.0005)	0.998 (0.997, 0.999), <0.001 (0.0005)
Female	0.852 (0.845, 0.860), <0.001 (0.003)	0.852 (0.845, 0.860), <0.001 (0.003)	0.810 (0.807, 0.813) <0.001 (0.001)	0.810 (0.807, 0.813) <0.001 (0.001)
Index of Multiple Deprivation 2015	1.021 (1.020, 1.021), <0.001 (0.0001)	1.021 (1.020, 1.021), <0.001 (0.0001)	1.021 (1.020, 1.021), <0.001 (0.0001)	1.021 (1.020, 1.021), <0.001 (0.0001)
Rural (v urban)	0.875 (0.862, 0.887), <0.001 (0.006)	0.875 (0.862, 0.887), <0.001 (0.006)	0.865 (0.857, 0.873), <0.001 (0.004)	0.865 (0.857, 0.873), <0.001 (0.004)
Ethnicity (%White)	1.0004 (1.0001, 1.0008), <0.002 (0.005)	1.0004 (1.0001, 1.0008), <0.002 (0.005)	0.999 (0.998, 0.999), <0.001 (0.0001)	0.999 (0.998, 0.999), <0.001 (0.0001)
Age (0-04)	*Reference Category*	*Reference Category*	*Reference Category*	*Reference Category*
Age (05-09)	0.988 (0.952, 1.026), <0.543 (0.019)	0.988 (0.952, 1.026), <0.541 (0.019)	0.792 (0.782, 0.803), <0.001 (0.005)	0.792 (0.782, 0.803), <0.001 (0.005)
Age (10-14)	0.720 (0.689, 0.752), <0.001 (0.016)	0.720 (0.689, 0.752), <0.001 (0.016)	0.566 (0.556, 0.576), <0.001 (0.005)	0.566 (0.556, 0.576), <0.001 (0.005)
Age (15-19)	0.653 (0.625, 0.682), <0.001 (0.015)	0.654 (0.626, 0.683), <0.001 (0.015)	0.520 (0.511, 0.530), <0.001 (0.005)	0.521 (0.511, 0.530), <0.001 (0.005)
Age (20-24)	0.688 (0.658, 0.718) <0.001 (0.015)	0.691 (0.661, 0.722) <0.001 (0.016)	0.488 (0.479, 0.497) <0.001 (0.005)	0.489 (0.479, 0.498) <0.001 (0.005)
Age (25-29)	0.553 (0.529, 0.577), <0.001 (0.012)	0.553 (0.529, 0.578), <0.001 (0.012)	0.409 (0.402, 0.417), <0.001 (0.004)	0.409 (0.402, 0.417), <0.001 (0.004)
Age (30-34)	0.583 (0.558, 0.609) <0.001 (0.013)	0.583 (0.559, 0.609) <0.001 (0.013)	0.438 (0.430, 0.446), <0.001 (0.004)	0.438 (0.430, 0.446), <0.001 (0.004)
Age (35-39)	0.665 (0.639, 0.693) <0.001 (0.013)	0.665 (0.639, 0.693) <0.001 (0.013)	0.531 (0.522, 0.540), <0.001 (0.004)	0.531 (0.522, 0.540), <0.001 (0.005)
Age (40-44)	0.914 (0.880, 0.949), <0.001 (0.018)	0.914 (0.880, 0.949), <0.001 (0.018)	0.719 (0.708, 0.730), <0.001 (0.006)	0.719 (0.708, 0.730), <0.001 (0.006)
Age (45-49)	1.265 (1.222, 1.309), <0.001 (0.22)	1.265 (1.222, 1.309), <0.001 (0.22)	1.000 (0.986, 1.014), <0.972 (0.007)	1.000 (0.986, 1.014), <0.974 (0.007)

Grigoroglou C, *et al. BMJ Open* 2020; 10:e036046. doi: 10.1136/bmjopen-2019-036046

Age (50-54)	1.759 (1.701, 1.817), <0.001 (0.030)	1.759 (1.701, 1.818), <0.001 (0.030)	1.388 (1.370, 1.407), <0.001 (0.010)	1.388 (1.370, 1.407), <0.001 (0.010)
Age (55-59)	2.467 (2.388, 2.550), <0.001 (0.041)	2.467 (2.387, 2.550), <0.001 (0.041)	1.909 (1.884, 1.934), <0.001 (0.013)	1.909 (1.884, 1.934), <0.001 (0.013)
Age (60-64)	3.414 (3.306, 3.525), <0.001 (0.056)	3.413 (3.306, 3.524), <0.001 (0.056)	2.671 (2.637, 2.706), <0.001 (0.017)	2.671 (2.637, 2.706), <0.001 (0.017)
Age (65-69)	4.590 (4.450, 4.733), <0.001 (0.072)	4.589 (4.450, 4.732), <0.001 (0.072)	3.838 (3.791, 3.886), <0.001 (0.024)	3.838 (3.791, 3.886), <0.001 (0.024)
Age (70-74)	6.942 (6.734, 7.157), <0.001 (0.109)	6.940 (6.732, 7.155), <0.001 (0.109)	5.667 (5.600, 5.736), <0.001 (0.035)	5.667 (5.599, 5.735), <0.001 (0.035)
Age (75-79)	9.808 (9.516, 10.108), <0.001 (0.151)	9.804 (9.512, 10.104), <0.001 (0.151)	7.873 (7.782, 7.965), <0.001 (0.047)	7.871 (7.780, 7.964), <0.001 (0.047)
Age (80-84)	13.380 (12.985, 13.787), <0.001 (0.205)	13.374 (12.979, 13.780), <0.001 (0.205)	10.714 (10.591, 10.839), <0.001 (0.063)	10.712 (10.589, 10.837), <0.001 (0.063)
Age (85plus)	18.448 (17.909, 19.003) <0.001 (0.279)	18.437 (17.898, 18.992) <0.001 (0.279)	14.363 (14.198, 14.529), <0.001 (0.084)	14.359 (14.195, 14.526), <0.001 (0.084)
2006	*Reference Year*	*Reference Year*	*Reference Year*	*Reference Year*
2007	-	-	0.947 (0.942, 0.952), <0.001 (0.003)	0.947 (0.942, 0.952), <0.001 (0.003)
2008	-	-	0.965 (0.959, 0.971), <0.001 (0.003)	0.965 (0.960, 0.971), <0.001 (0.003)
2009	-	-	0.929 (0.923, 0.934), <0.001 (0.003)	0.929 (0.923, 0.934), <0.001 (0.003)
2010	-	-	0.933 (0.927, 0.939), <0.001 (0.003)	0.933 (0.927, 0.939), <0.001 (0.003)
2011	-	-	0.900 (0.894, 0.906), <0.001 (0.003)	0.900 (0.894, 0.906), <0.001 (0.003)
2012	-	-	0.917 (0.910, 0.923), <0.001 (0.003)	0.916 (0.910, 0.923), <0.001 (0.003)
2013	-	-	0.893 (0.887, 0.899), <0.001 (0.003)	0.893 (0.887, 0.899), <0.001 (0.003)
2014	-	-	0.926 (0.921, 0.932), <0.001 (0.003)	0.927 (0.921, 0.932), <0.001 (0.003)
2015	-	-	0.930 (0.924, 0.936), <0.001 (0.003)	0.930 (0.924, 0.936), <0.001 (0.003)

Grigoroglou C, *et al. BMJ Open* 2020; 10:e036046. doi: 10.1136/bmjopen-2019-036046

Model intercept	0.0034 (0.0029, 0.0040), <0.001 (0.0002)	0.0035 (0.0029, 0.0041), <0.001 (0.0003)	0.004 (0.003, 0.004), <0.001 (0.0001)	0.004 (0.003, 0.004), <0.001 (0.0001)

Table S4 - Effect of QOF Population Achievement on age-adjusted hospital admissions for QOF incentivised ACSCs in 2015/16 [a,b,c,]

% Population achievement	-8.478 (-11.102, -5.854), <0.001 (1.338)
Index of Multiple Deprivation 2015	21.478 (21.113, 21.862), <0.001 (0.191)
Rural (v urban)	-90.872 (-106.159, -75.585), <0.001 (7.799)
% Population White	0.622 (0.302, 0.941), <0.001 (0.163)
Constant	1117.832 (900.384, 1335.280) <0.001 (110.940)
Adjusted R^2	0.3199

a A total of 32 844 LSOAs (observations)

b Adjusted R2 = 32.03%.

c Coefficients can be interpreted as rates of change, for example, A non-urban LSOA would correspond to an age-adjusted admission rate change of −90.9, compared with an urban LSOA.

Figure S3: Age-adjusted hospital admissions for QOF incentivised conditions at the LSOA level, 2015/16.

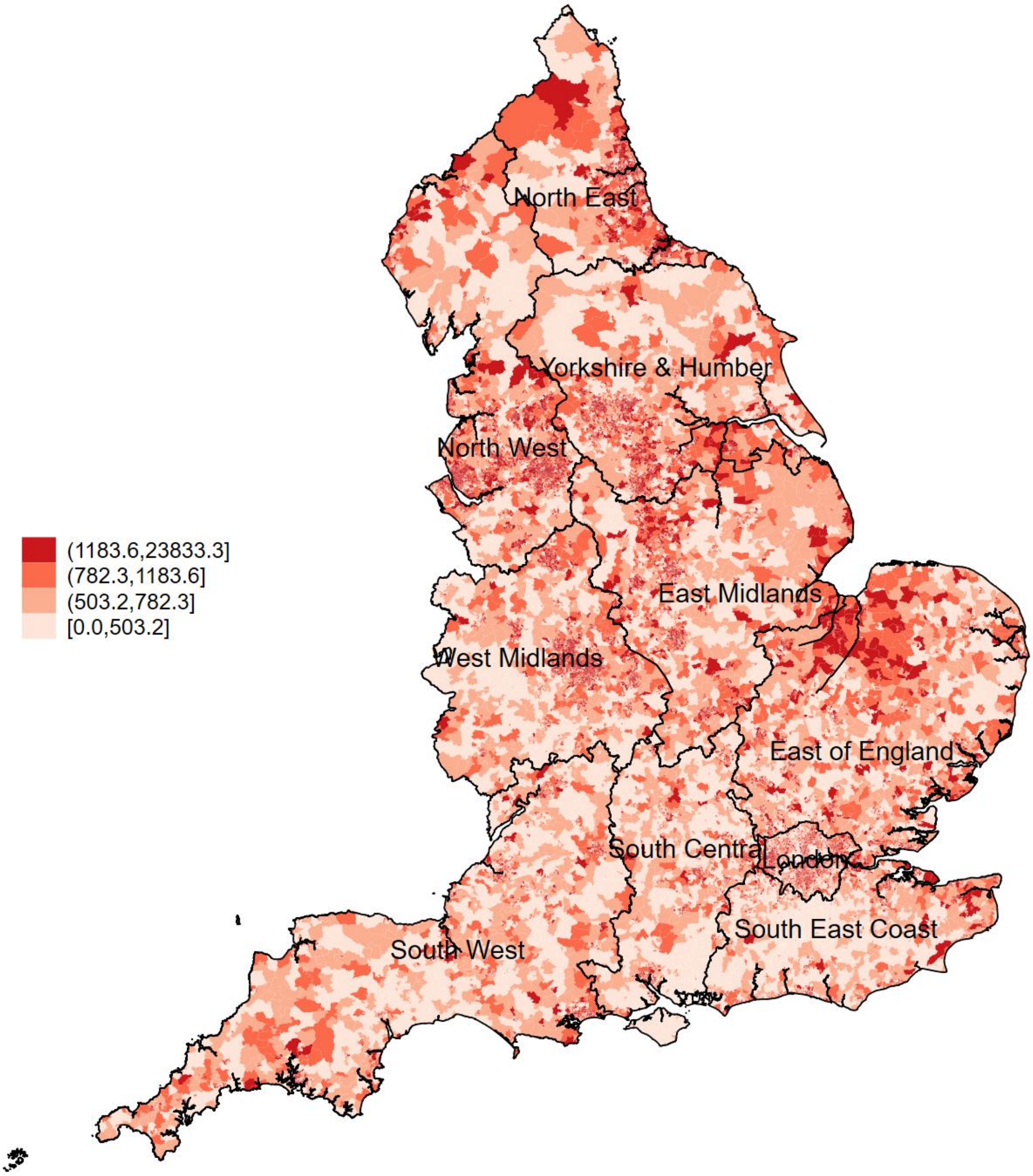

Grigoroglou C, *et al. BMJ Open* 2020; 10:e036046. doi: 10.1136/bmjopen-2019-036046

Regional spatial maps of hospital admissions for QOF incentivised ACSCs at the LSOA level, 2015/16.

Figure S3: North East

Grigoroglou C, *et al. BMJ Open* 2020; 10:e036046. doi: 10.1136/bmjopen-2019-036046

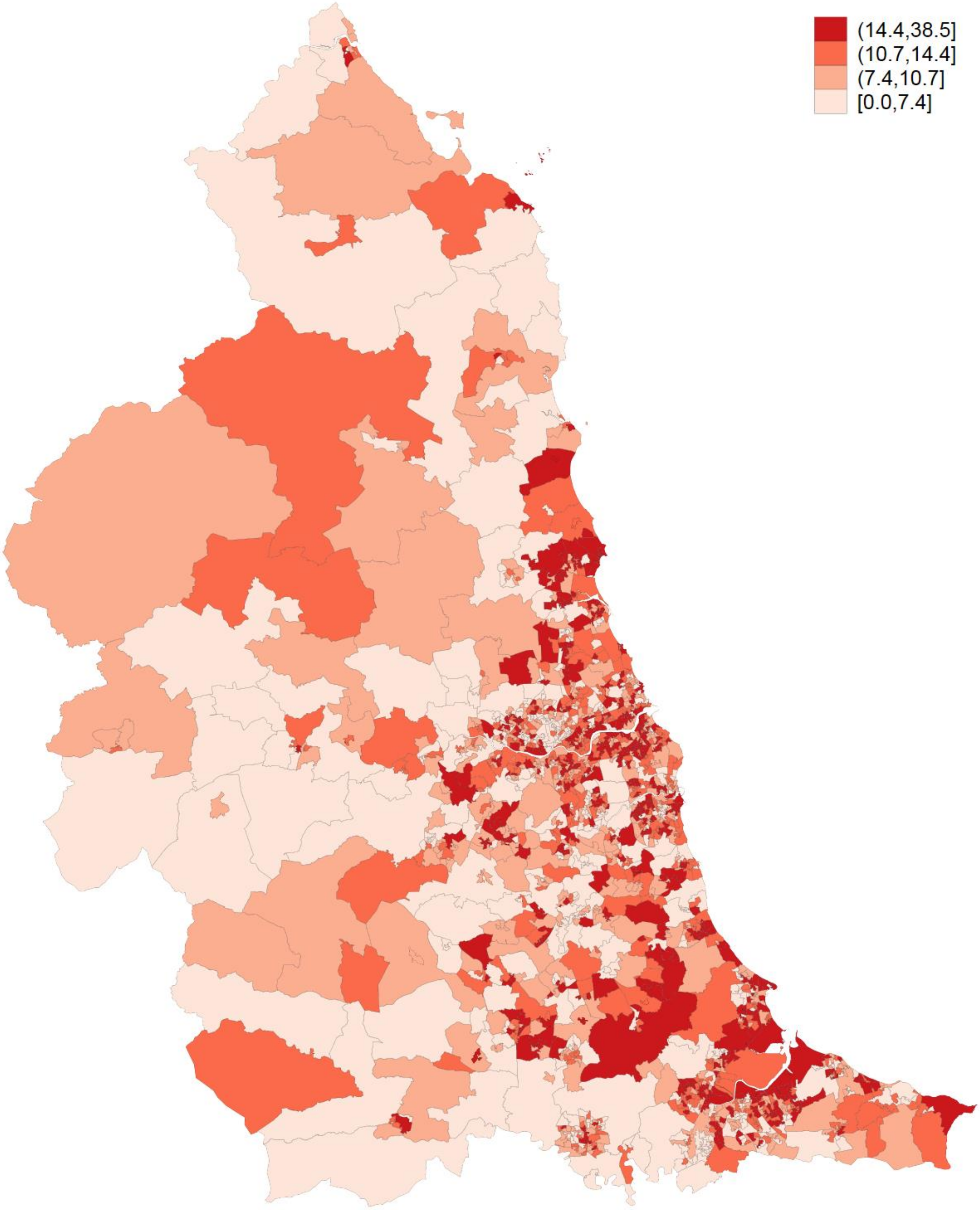

Figure S4: North West

Grigoroglou C, *et al. BMJ Open* 2020; 10:e036046. doi: 10.1136/bmjopen-2019-036046

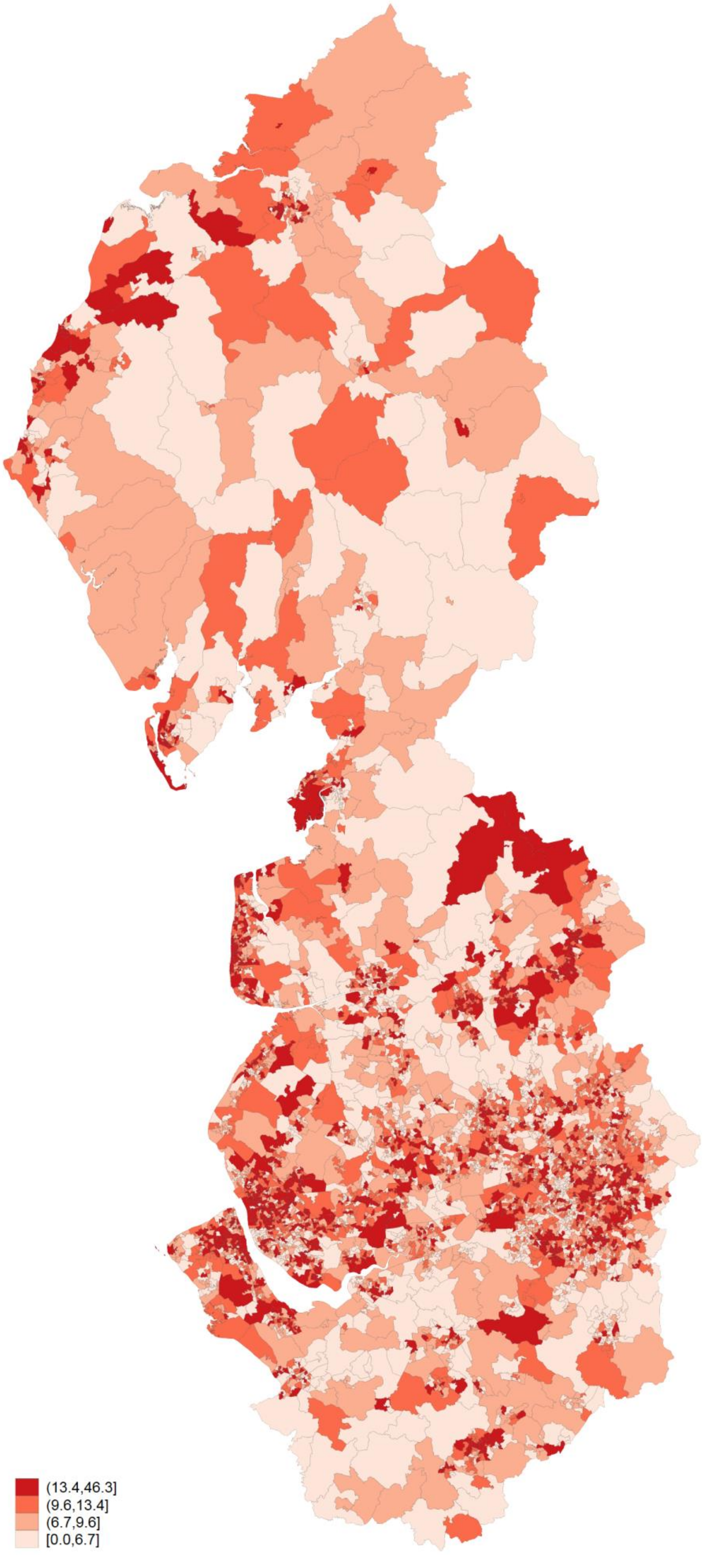

Figure S5: Yorkshire & Humber

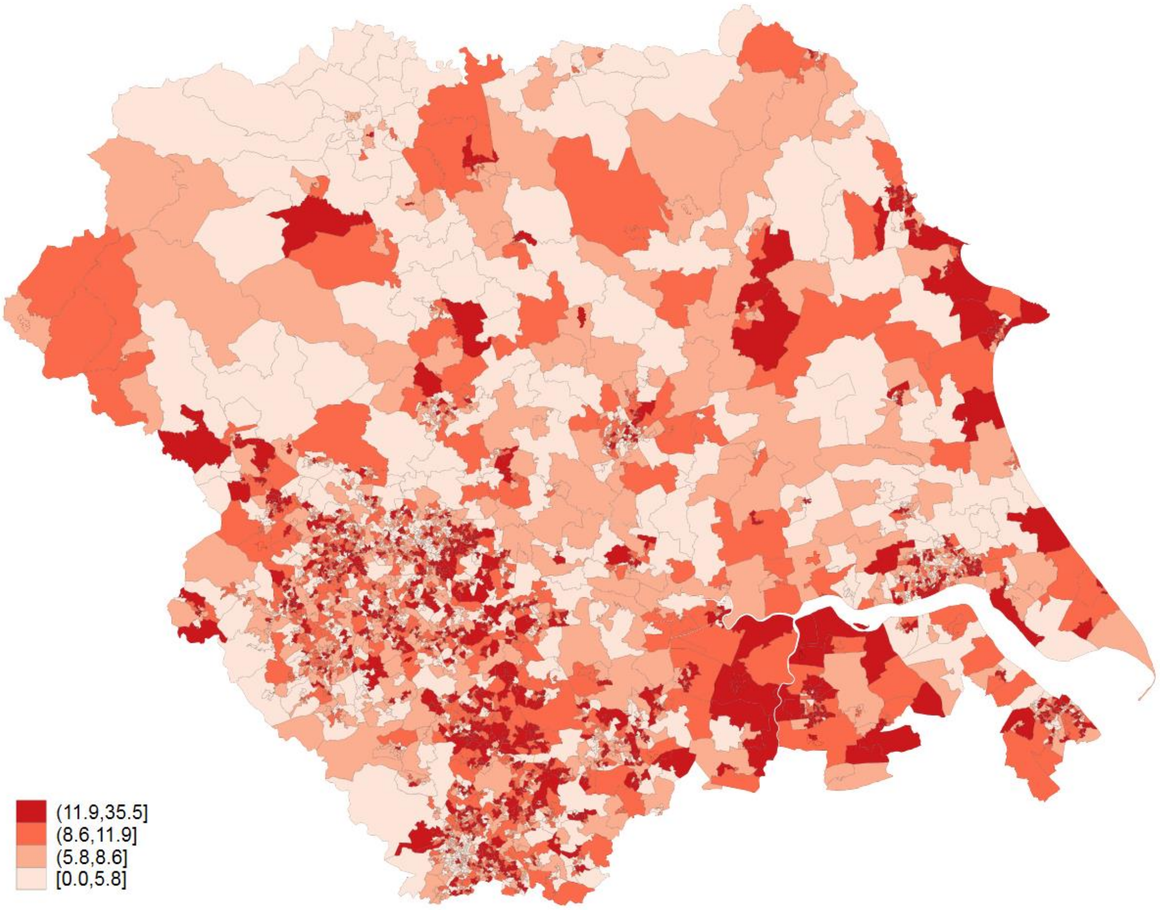
(11.9,35.5]
(8.6,11.9]
(5.8,8.6]
[0.0,5.8]

Figure S6: East Midlands

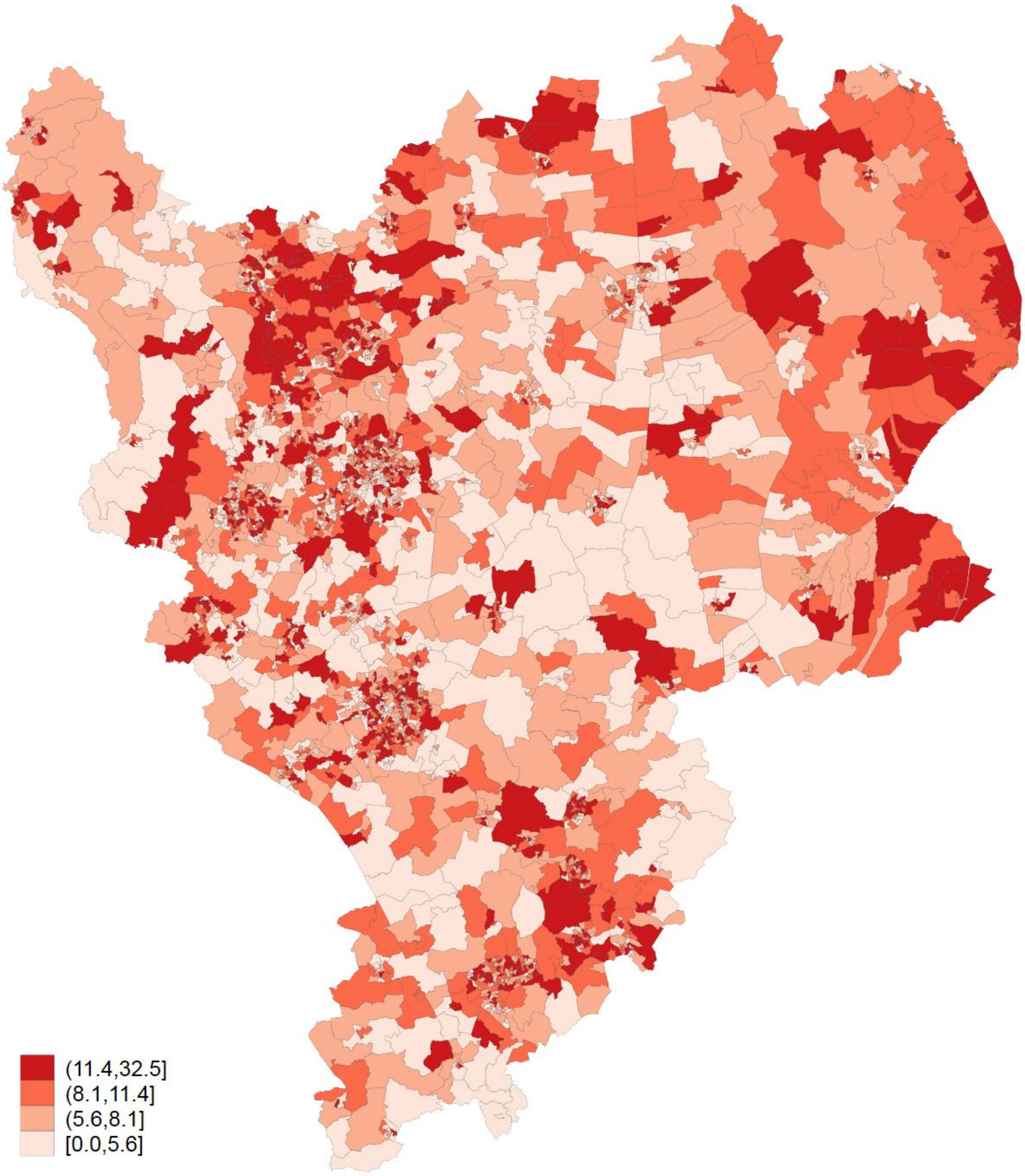

Grigoroglou C, *et al. BMJ Open* 2020; 10:e036046. doi: 10.1136/bmjopen-2019-036046

Figure S7: West Midlands

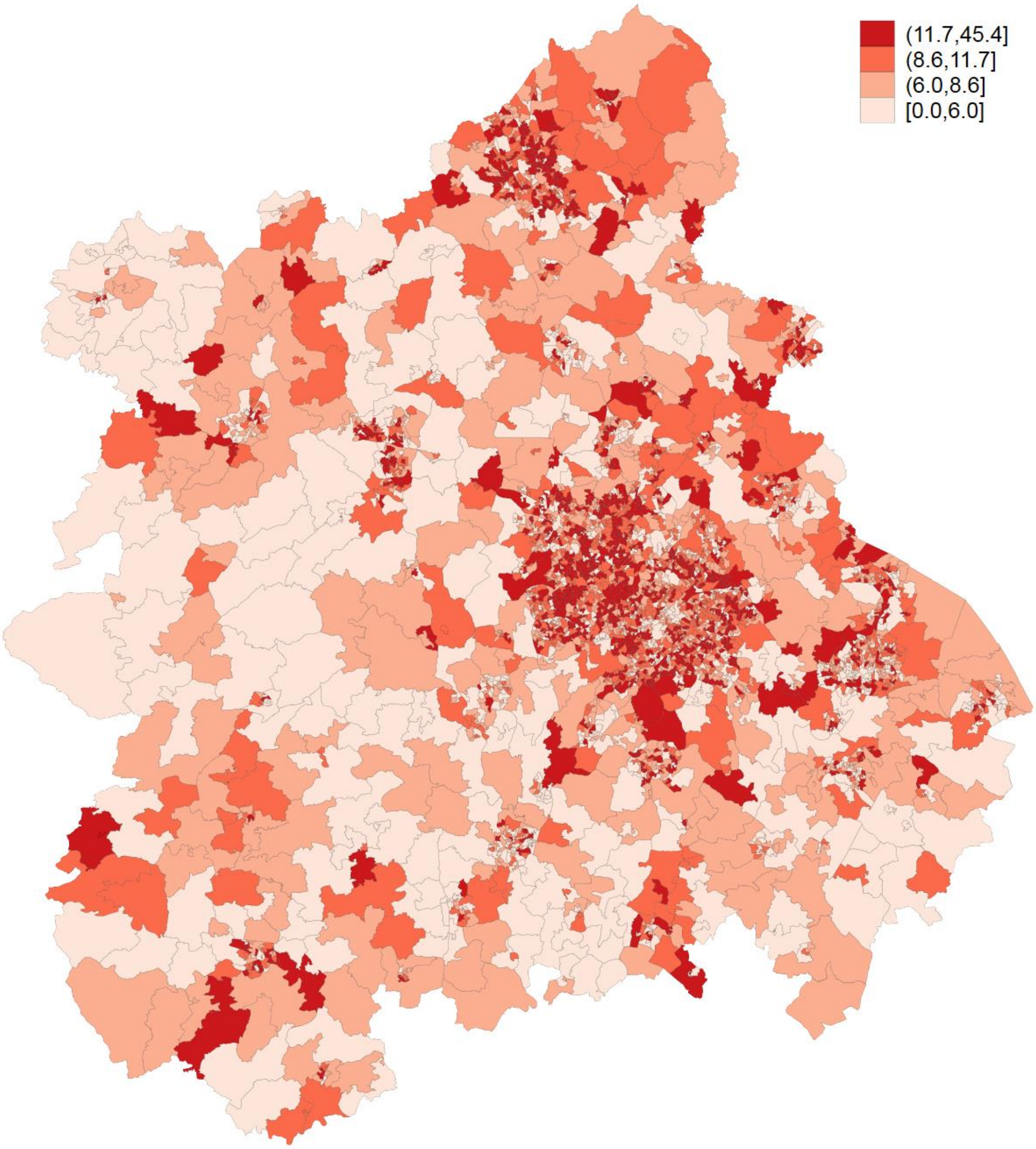

Figure S8: East of England

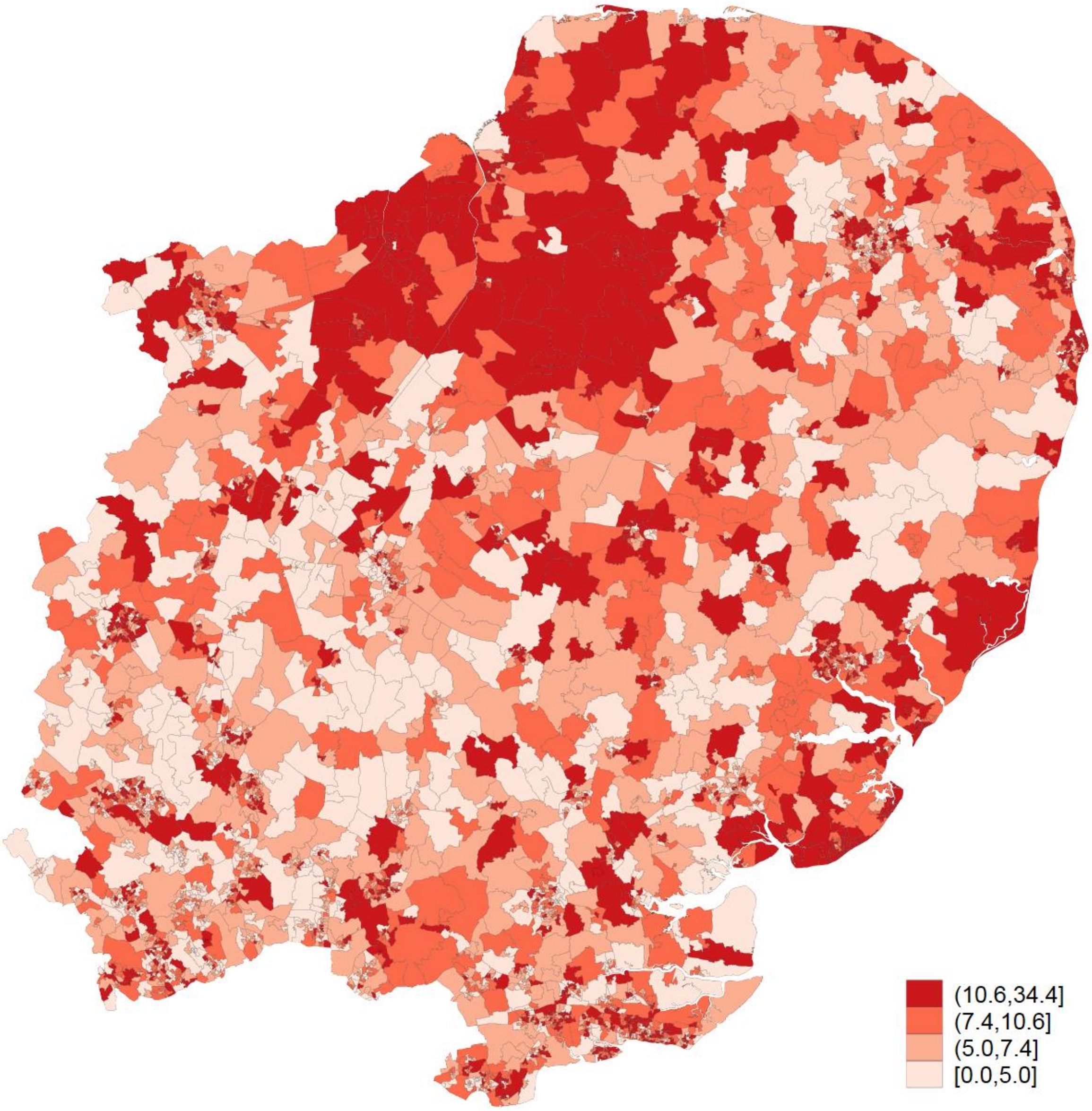

Figure S9: London

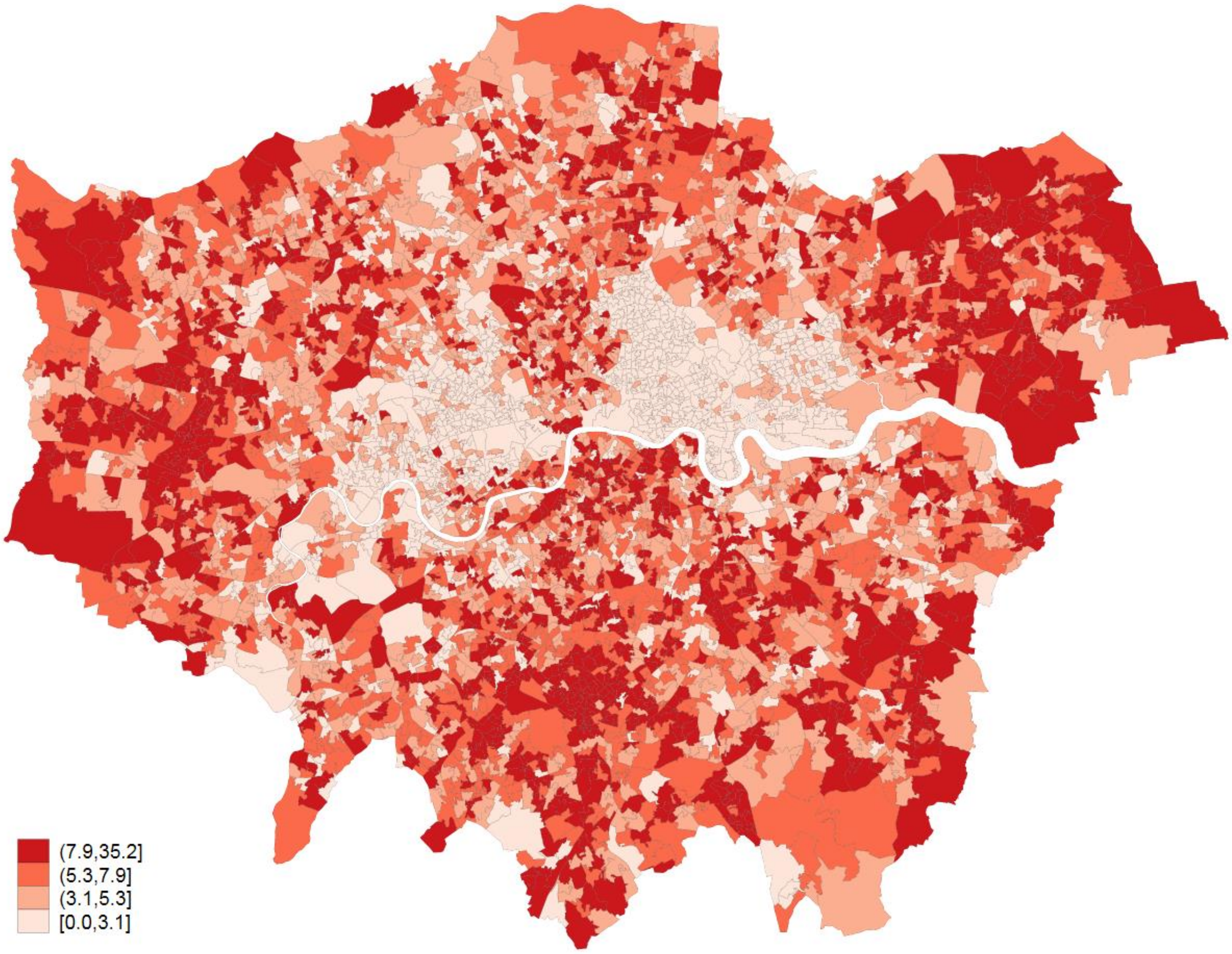

Figure S10: South East Coast

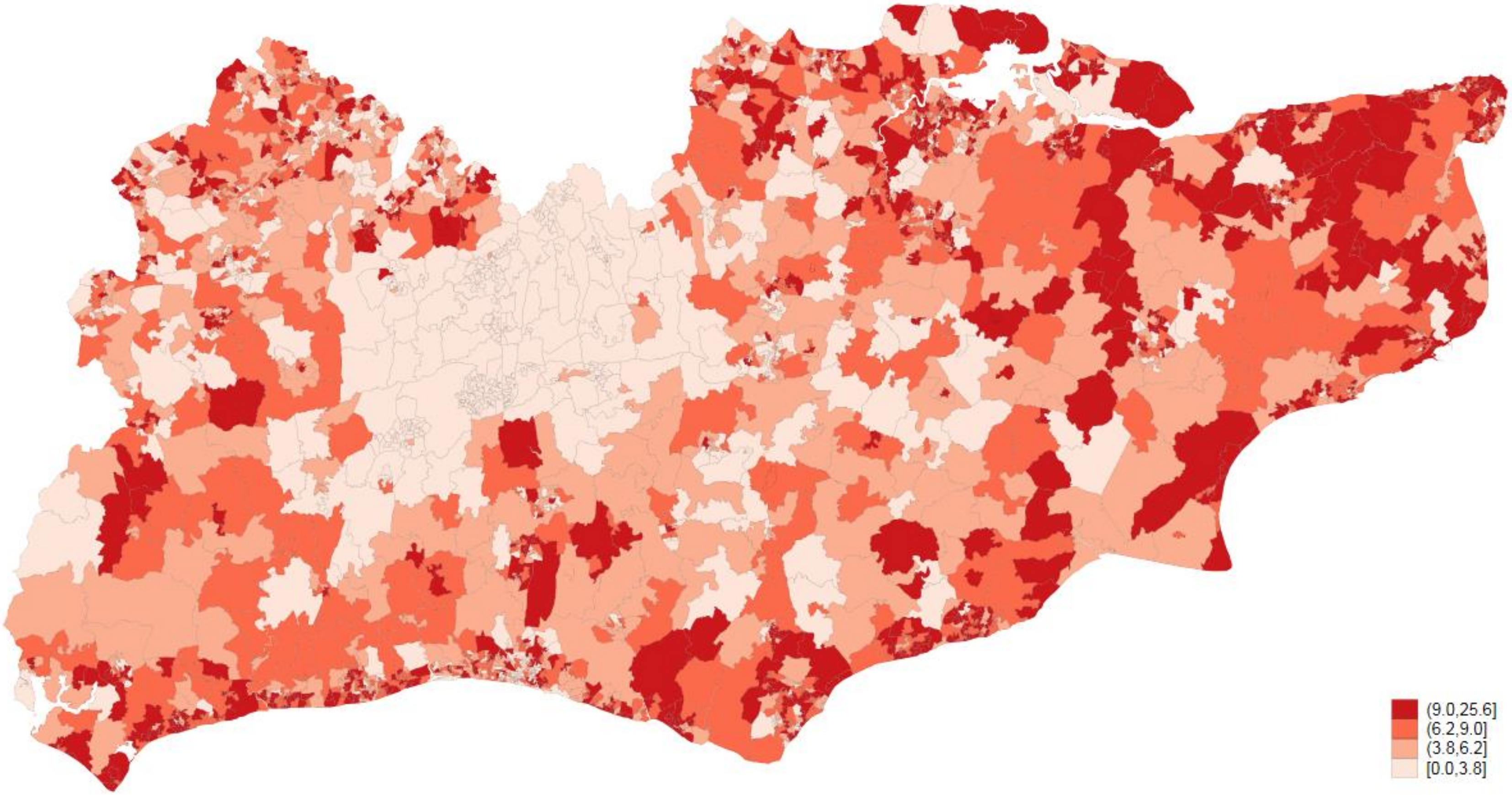

Figure S11: South Central

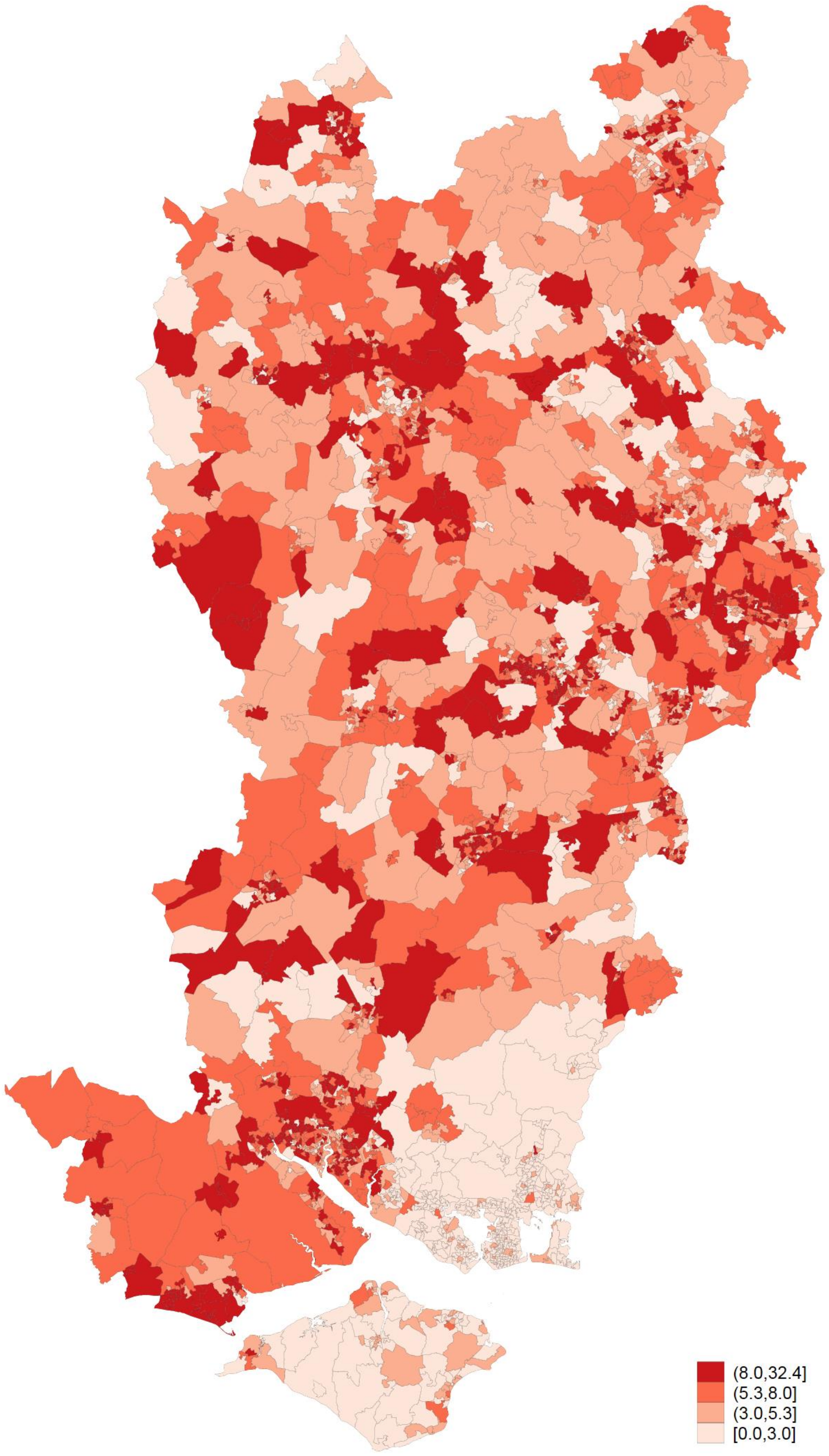

Figure S12: South West

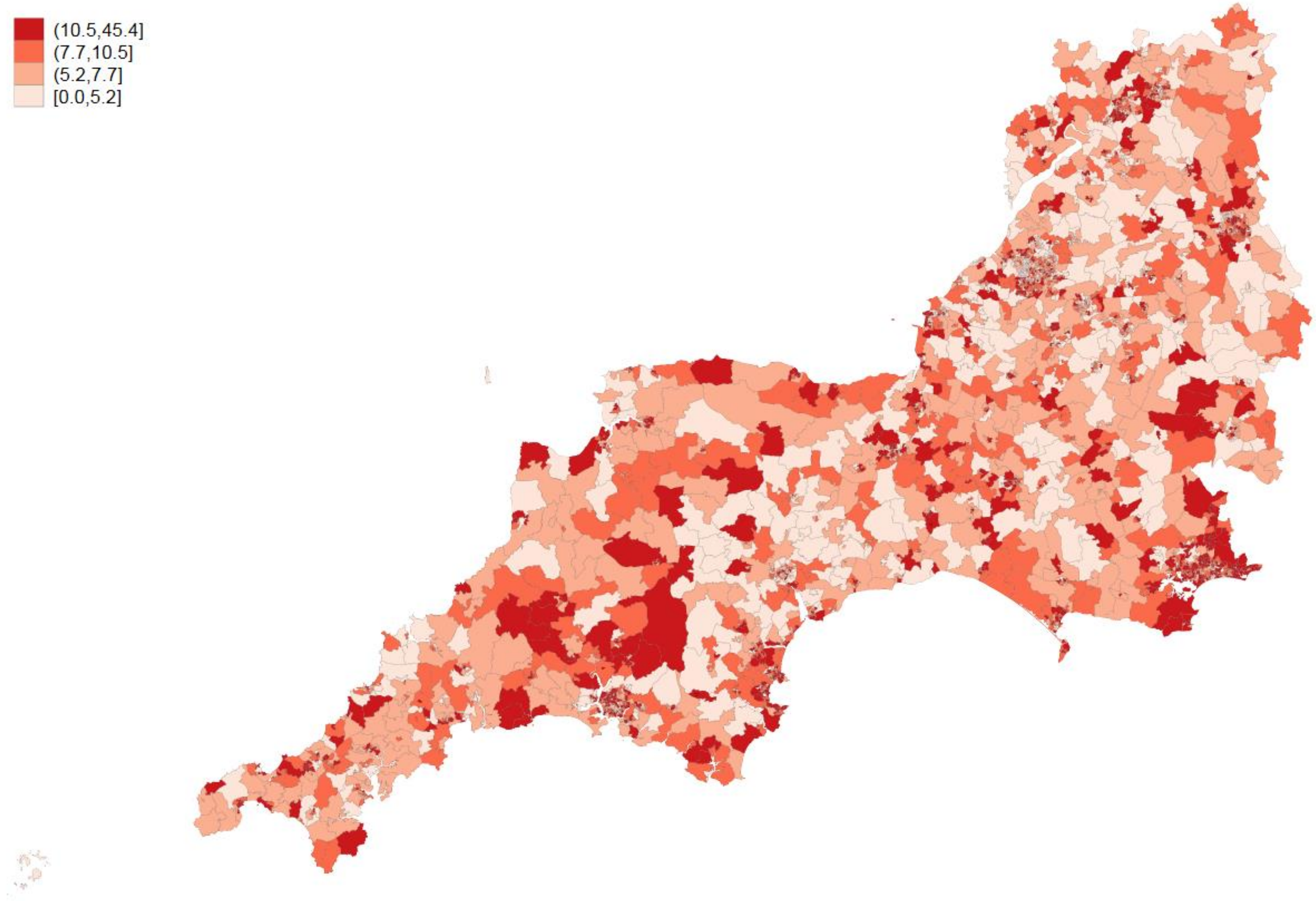

Grigoroglou C, *et al. BMJ Open* 2020; 10:e036046. doi: 10.1136/bmjopen-2019-036046

Regional Spatial Maps of Population Achievement (PAoval) for incentivised ACSCs at the LSOA level, 2015/16.

Figure S13: North East

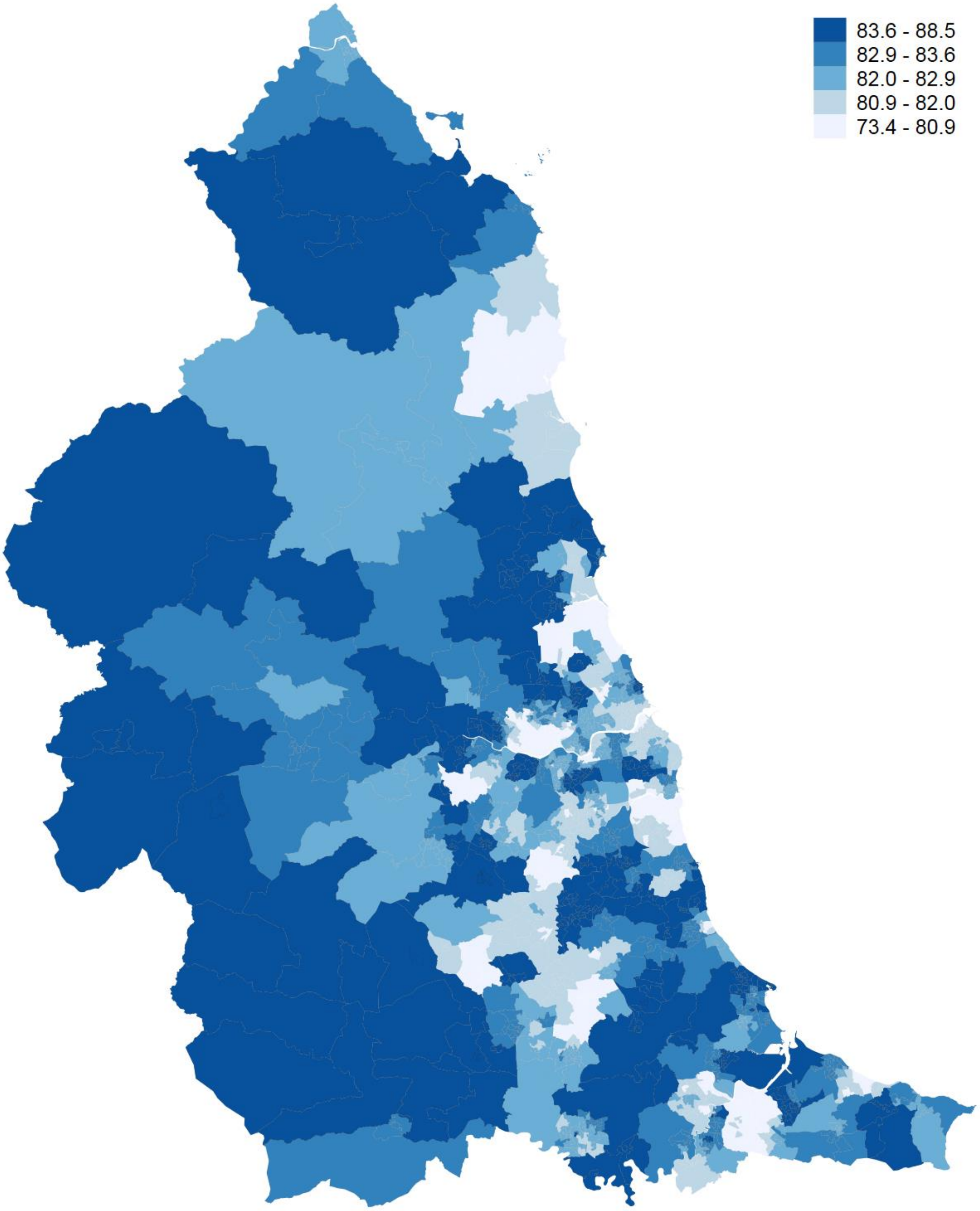

Figure S14: North West

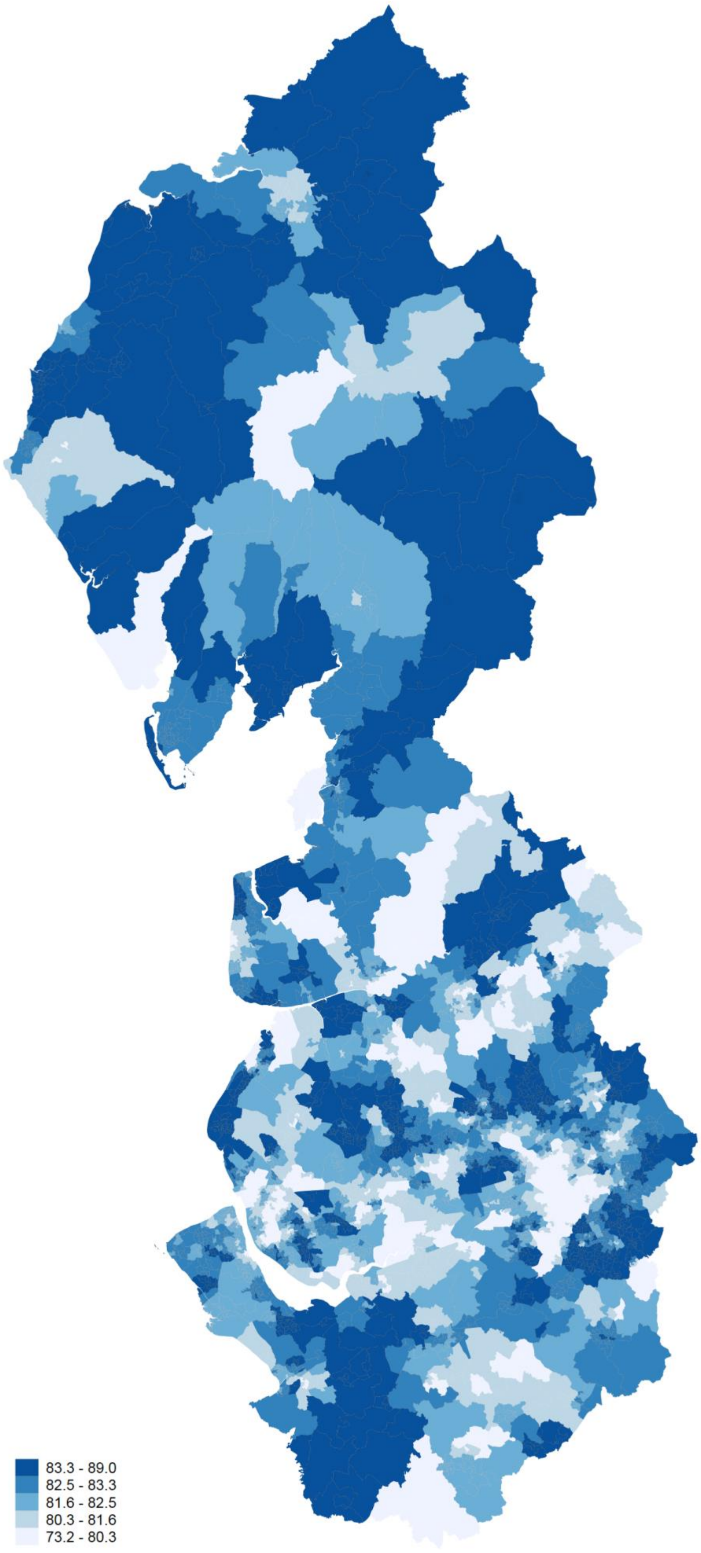

t

Figure S15: Yorkshire & Humber

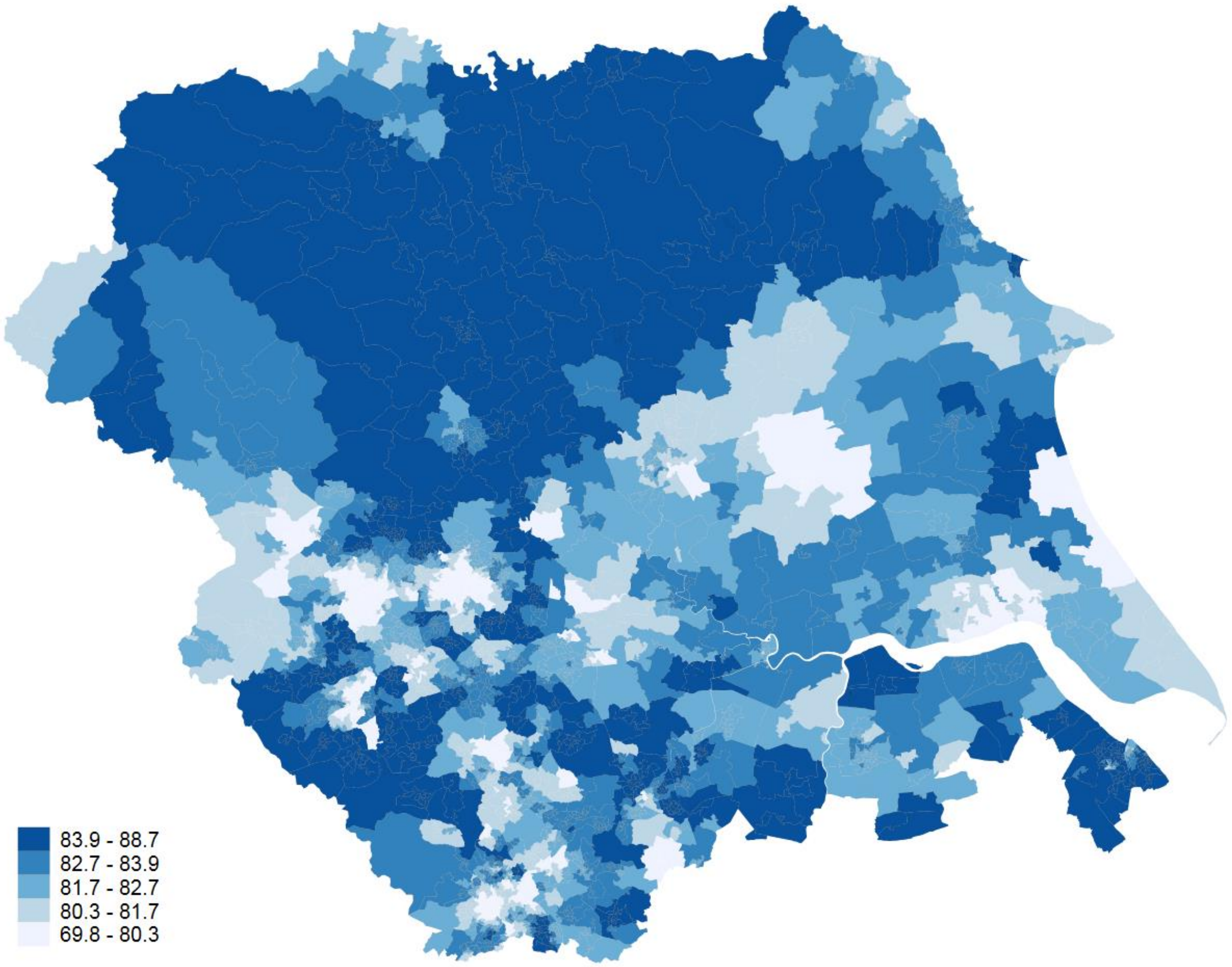

Figure S16: East Midlands

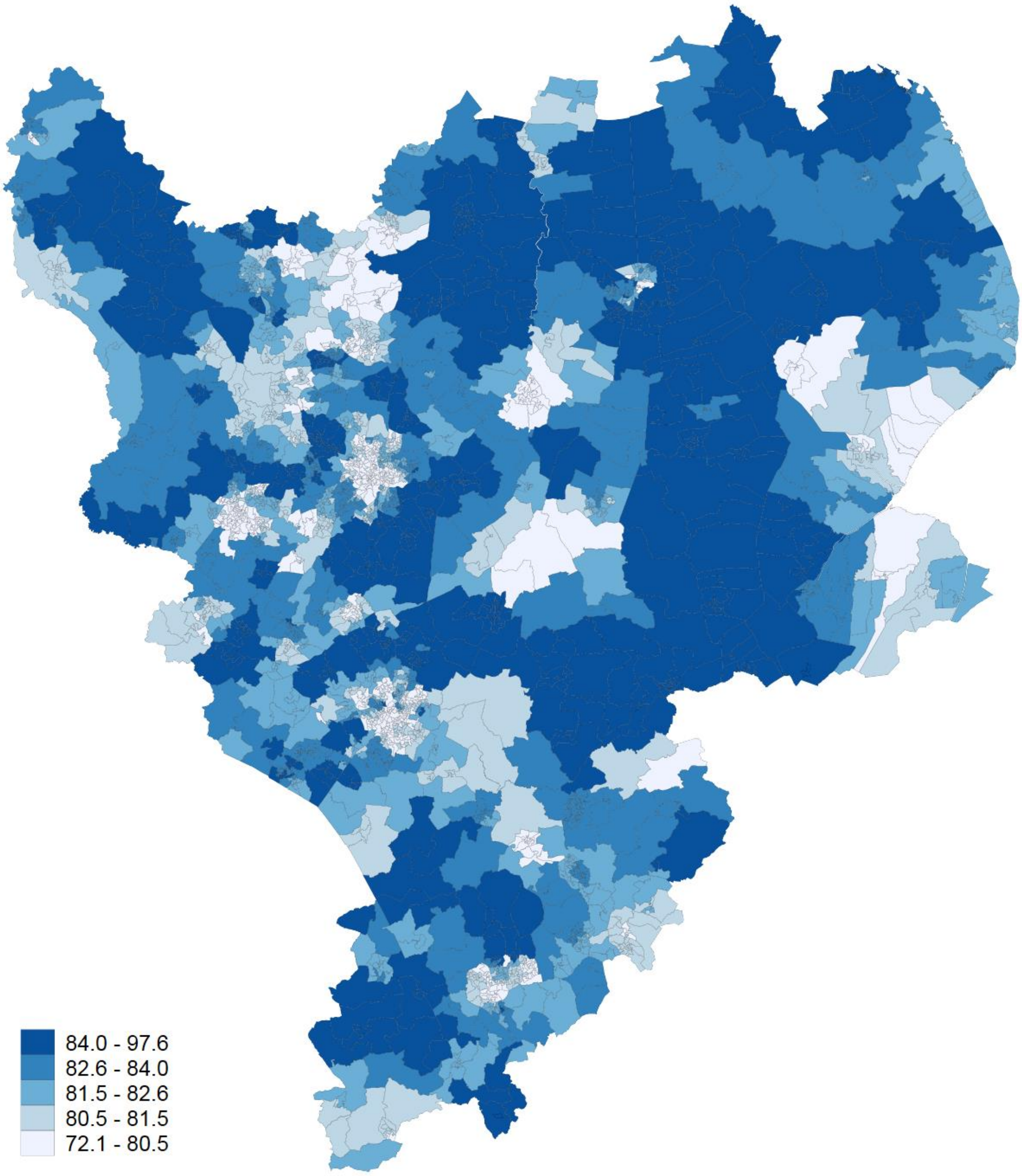

Grigoroglou C, *et al. BMJ Open* 2020; 10:e036046. doi: 10.1136/bmjopen-2019-036046

Figure S17: West Midlands

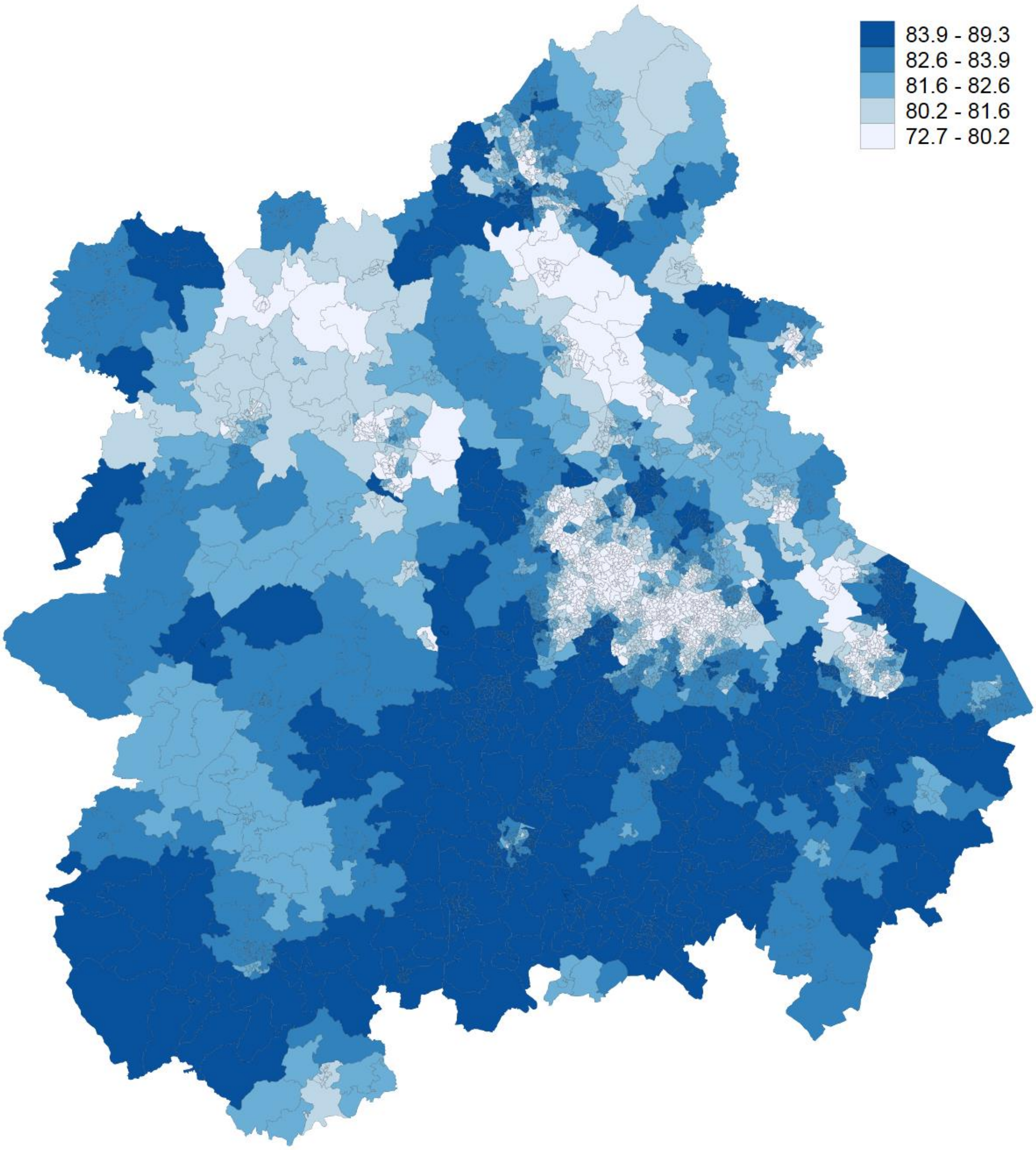

Figure S18: East of England

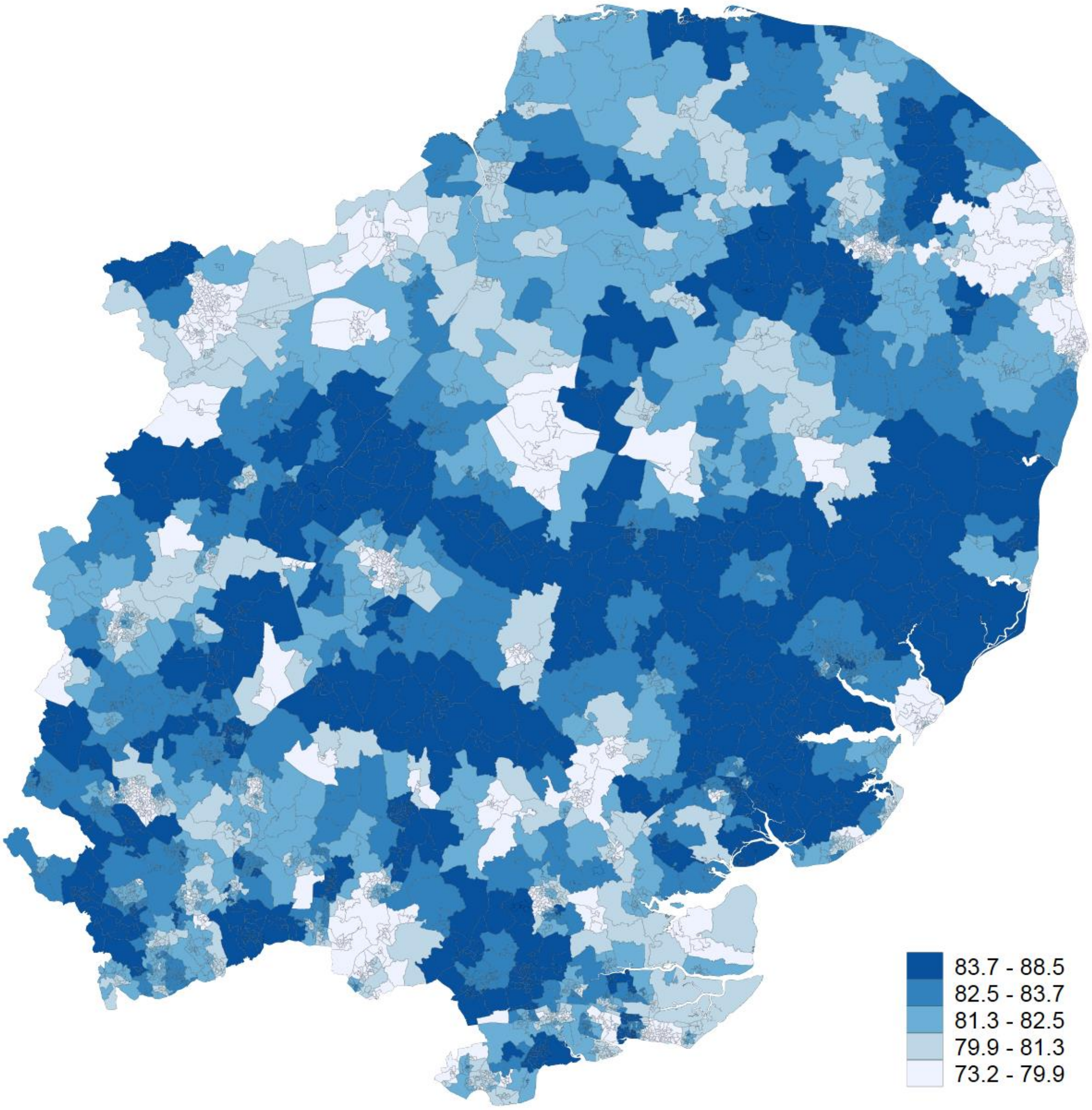

Grigoroglou C, *et al. BMJ Open* 2020; 10:e036046. doi: 10.1136/bmjopen-2019-036046

Figure S19: London

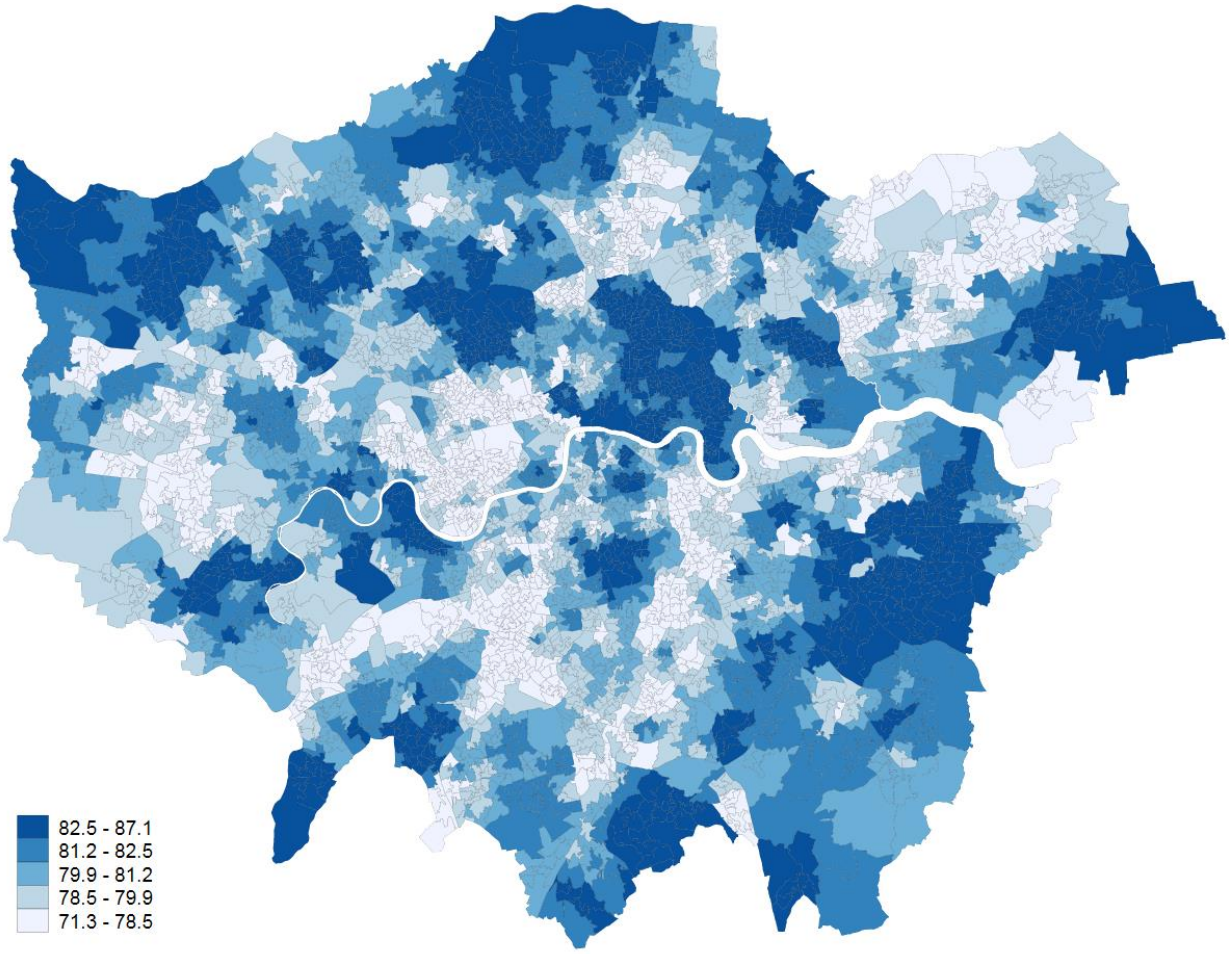

Grigoroglou C, *et al. BMJ Open* 2020; 10:e036046. doi: 10.1136/bmjopen-2019-036046

Figure S20: South East Coast

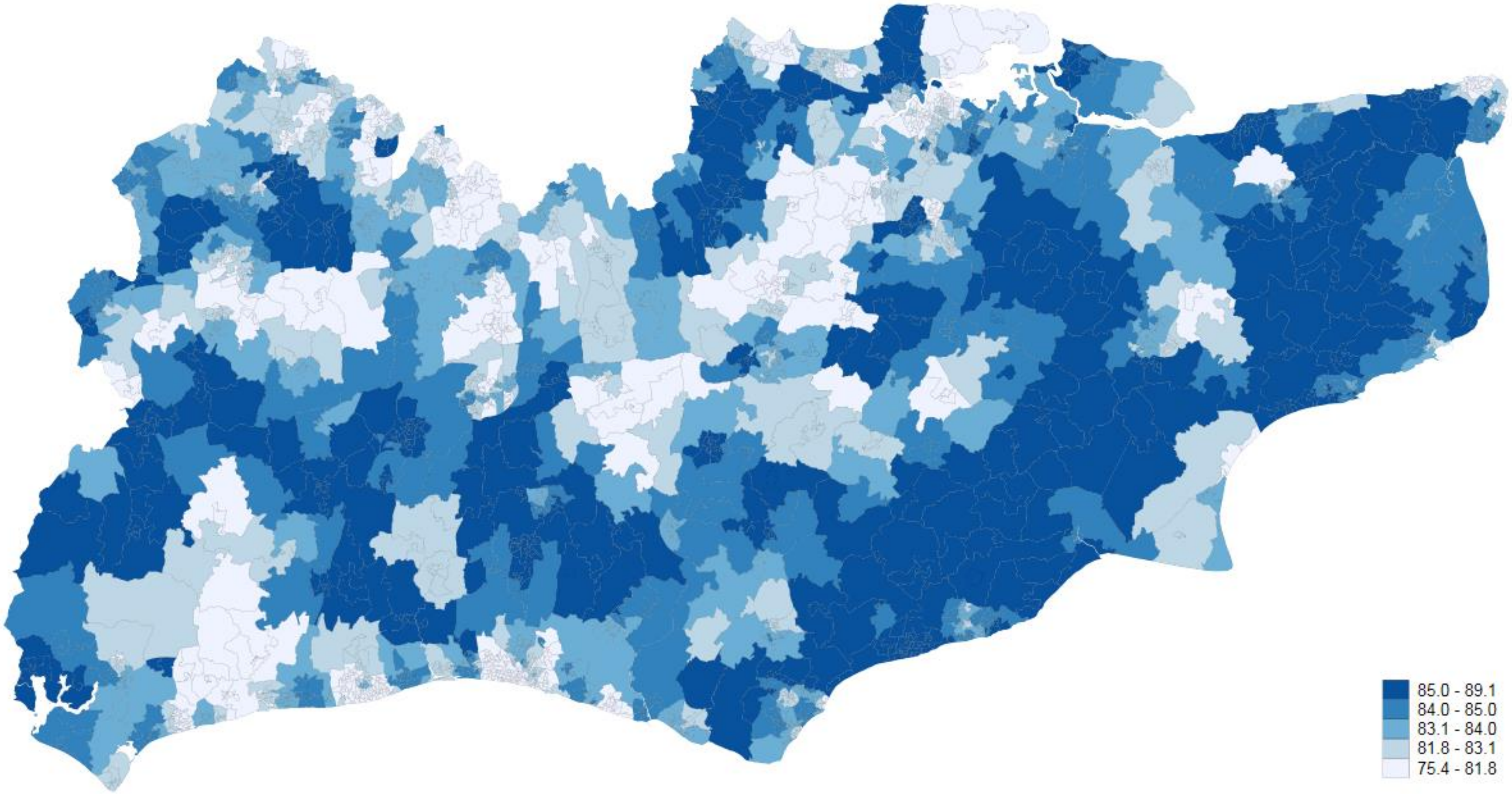

Grigoroglou C, *et al. BMJ Open* 2020; 10:e036046. doi: 10.1136/bmjopen-2019-036046

Figure S21: South Central

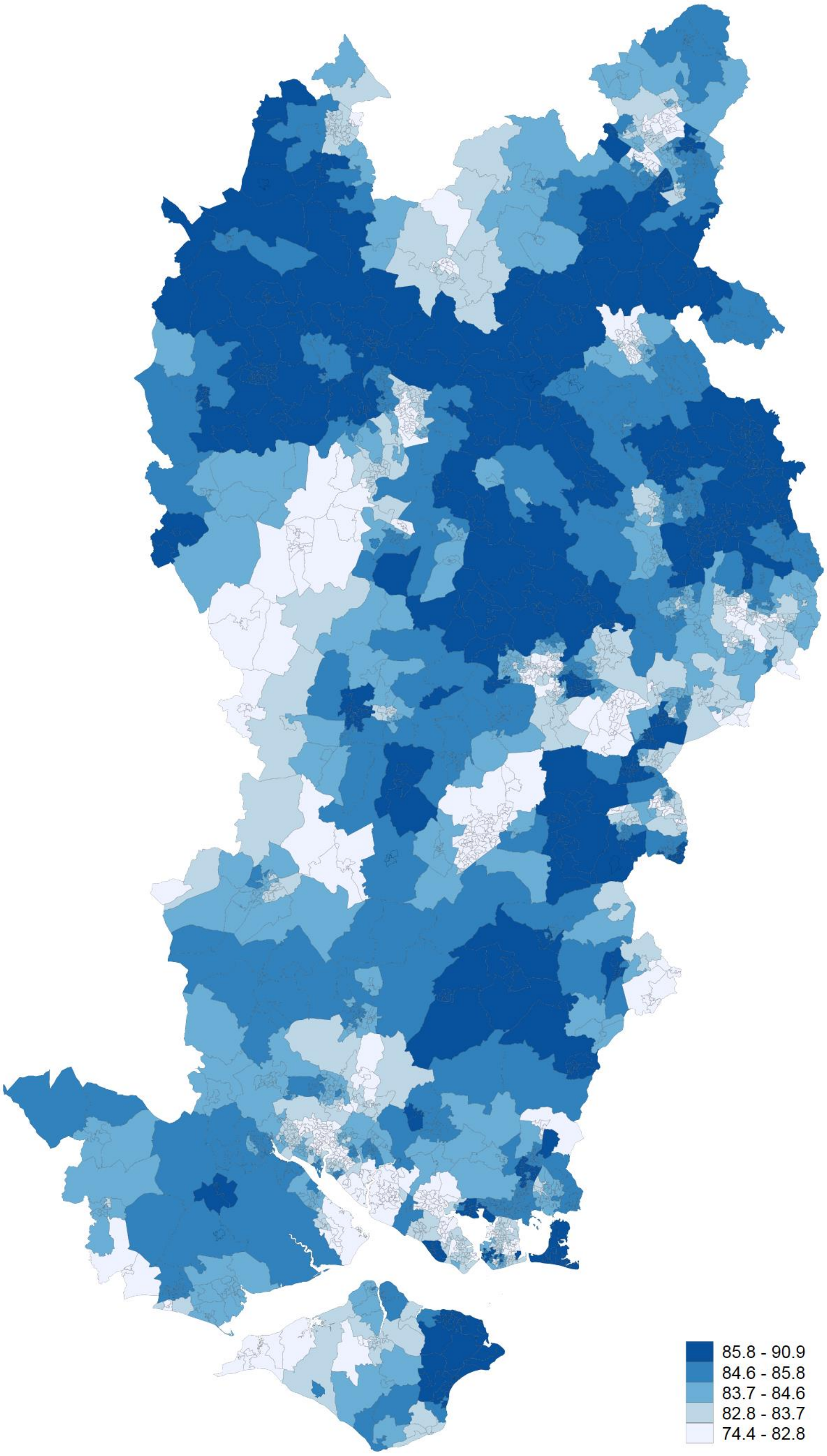

Figure S22: South West

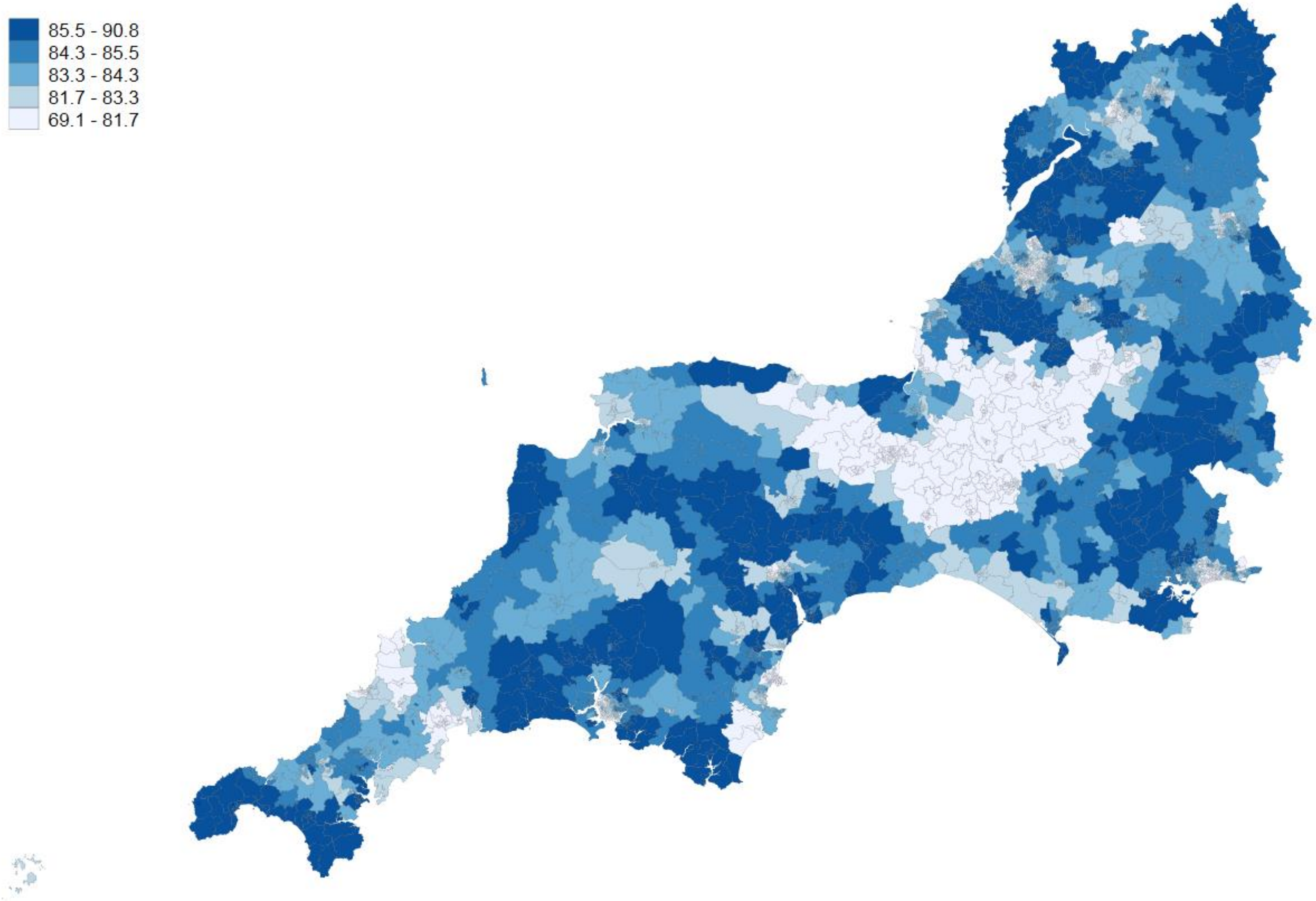

Grigoroglou C, *et al. BMJ Open* 2020; 10:e036046. doi: 10.1136/bmjopen-2019-036046

References

1. Purdy S, Griffin T, Salisbury C, Sharp D. Ambulatory care sensitive conditions: terminology and disease coding need to be more specific to aid policy makers and clinicians. *Public Health* 2009;123(2):169-73.
2. Dusheiko M, Doran T, Gravelle H, Fullwood C, Roland M. Does higher quality of diabetes management in family practice reduce unplanned hospital admissions? *Health Serv Res* 2011;46(1 Pt 1):27-46.
3. Department of Communities and Local Government. The English Indices of Deprivation 2015: Technical Report. 2015.
4. Office for National Statistics. Changes to output areas and super output areas in England and Wales, 2001 to 2011. 2012.
5. Bibby P SJ. Developing a new classification of urban and rural areas for policy purposes—the methodology. DEFRA. 2004.
6. Office for National Statistics. Super output area mid-year population estimates for England and Wales, mid-2011 (census based). 2015.
7. Office for National Statistics. Open Geography Portal. 2013b.
8. Office for National Statistics. Nomis official labour market statistics. 2013a.
9. NHS Digital. Attribution dataset GP registered populations. 2016.
10. Office for National Statistics. Open Geography Portal. 2013. https://geoportal.statistics.gov.uk/geoportal/
11. SHP2DTA: Stata module to converts shape boundary files to Stata datasets [program]. S456718 version: Boston College Department of Economics, 2006.
12. Office for National Statistics. Ordnance Survey. A guide to coordinate systems in Great Britain. 2013:43. www.ordnancesurvey.co.uk/docs/support/guide-coordinate-systems-great-britainpdf.
